# Unsaid thoughts: Thinking in the absence of verbal logical connectives

**DOI:** 10.3389/fpsyg.2022.962099

**Published:** 2022-09-28

**Authors:** David J. Lobina, Josep Demestre, José E. García-Albea, Marc Guasch

**Affiliations:** ^1^Psycholinguistics Research Group, Universitat Rovira i Virgili, Tarragona, Spain; ^2^Department of Psychology and Research Center for Behavior Assessment, Universitat Rovira i Virgili, Tarragona, Spain; ^3^Department of Psychology, Universitat Rovira i Virgili, Tarragona, Spain

**Keywords:** language, thought, logical connectives, lexicalisation, learnability

## Abstract

Combining two thoughts into a compound mental representation is a central feature of our verbal and non-verbal logical abilities. We here approach this issue by focusing on the contingency that while natural languages have typically lexicalised only two of the possible 16 binary connectives from formal logic to express compound thoughts—namely, the coordinators *and* and *or*—some of the remainder appear to be entertainable in a non-verbal, conceptual representational system—a *language of thought*—and this suggests a theoretical split between the “lexicalisation” of the connectives and the “learnability” of invented words corresponding to unlexicalised connectives. In a *visual world* experiment aimed at tracking comprehension-related as well as reasoning-related aspects of the capacity to represent compound thoughts, we found that participants are capable of learning and interpreting a made-up word standing for logic's NAND operator, a result that indicates that unlexicalised logical connectives are not only conceptually available, but can also be mapped onto new function words, as in the case of coordinators, or connectives, a class of words that do not usually admit new coinages.

## 1. Introduction

A core feature of cognition is the ability to combine two propositions (or thoughts) into complex mental representations (Frege, 1963; Fodor, 1975), and a principal way to examine this capacity is by employing the tools of formal logic, a field of study that has informed our understanding of human reasoning since antiquity and which remains prominent in contemporary cognitive science (Braine and O'Brien, 1998; Johnson-Laird and Yang, 2008). The input from logic has been especially fruitful in experimental work on how we represent, and reason with, compound propositions, including the complementary ability to generate further thoughts from such propositions—i.e., to draw conclusions from complex propositions (Paris, 1973; Braine and Rumain, 1981).

Much of the evidence has come from studies using linguistic representations of propositions, given that complex propositions can be often expressed linguistically through so-called coordinators (Haspelmath, 2004), as in the compound sentence *the triangle is yellow AND/OR the square is blue*, where each clause (*the triangle is yellow, the square is blue*) constitutes a proposition and the coordinators “and/or” function as the sentential operators, or connectives, from logic. This kind of approach has necessarily often strayed into the rather thorny question of how the human capacity for language relates to the ability to think and reason, and as we shall argue here, the study of language's logical connectives can indeed highlight both verbal and non-verbal reasoning processes, as well as their interaction.

The use of linguistic representations as a proxy to probe how we reason with compound propositions can give rise to some complications, however. A notable problem stems from the nature of language itself and of linguistic communication in particular: compound sentences are underlain by properties of various kinds, and these can all play a role in the interpretation and use of these sentences in actual exchanges. Simplifying somewhat, these features can be split into two types, the linguistic and the non-linguistic. Relevant linguistic properties include the syntax of a sentence as well as the semantic and pragmatic information sentences codify, while on the side of non-linguistic properties we really ought to note that compound sentences can give rise to various *rules of inference*, logical rules that are not explicitly encoded in the sentences themselves but which can have an effect on how such sentences are comprehended and used. Unsurprisingly, and given the way in which these properties can interrelate, an experimental investigation evaluating how compound sentences are processed, and indeed logically interpreted, needs to pay especial attention to these features.

On the linguistic side of things, compound sentences very often appear to behave in ways that diverge from what is the case in logic (Klinedinst and Rothschild, 2012); conjunction *and*, for instance, can signal much more than a simple union of propositions, which is what logic mandates (e.g., it can mark a temporal, and even causal, relationship between two clauses, as in *the bomb exploded and the car was destroyed*). The consensus in most of the literature, however, is that the meaning—the semantics—of the relevant compound sentences (and, thus, of the corresponding linguistic coordinators) is analogous to the meanings logicians assign to compound propositions and connectives in terms of truth tables, with the corollary that the non-logical uses these complex sentences exhibit in language use may be the result of diverse pragmatic processes (presuppositions, implicatures, etc.; some semantic theories might not compartmentalise these properties in quite this way, though). To be more precise, this way of looking at the issue is based on the contingency that for the most part the pragmatic effects that arise in language comprehension can be nullified in various ways, thereby unearthing the logical meaning of the applicable compound sentences. Among other ways, pragmatic effects can be cancelled by the context or the addition of further linguistic material (Grice, 1989), they can be blocked by specific syntactic configurations (Chierchia, 2006), and some of them can even be controlled for in an experimental setting (Lobina et al., 2022), the latter the approach we used in this study.

Thus, the meaning of language's *and* would correspond to the truth table of logic's conjunction (∧, shown in column 3 of Table 1)—viz., a conjunctive compound sentence is only true when both clauses are true. A similar situation applies to language's *or*, though the state of affairs in this case is more intricate. Even though language's *or* often receives an “either/or” reading in linguistic exchanges, which would entail that its meaning corresponds to logic's exclusive disjunction (∨_*e*_; column 6)—i.e., a disjunctive sentence would be true only if either one of the two clauses is true—its actual, default meaning, as ascertained both empirically and through various linguistic tests, is in fact intrinsically inclusive (Gazdar and Pullum, 1976; Gazdar, 1979; Chevallier et al., 2008). That is, language's *or* specifies an “P or Q, or both” interpretation, and therefore its (semantic) meaning more properly matches the truth table of logic's inclusive disjunction (∨; column 5)—a disjunctive compound sentence is true if one of the two clauses is true as well as when both clauses are true (it is important to note that the truth table of exclusive disjunction constitutes a subset of the truth table of inclusive disjunction, both in logic and in language; this will be an important issue later on). The either/or reading seemingly so common in linguistic exchanges is argued to be the result of an “exclusivity implicature” hearers tend to compute in real-time communication.^1^

**Table 1:** Logic's truth tables.

**P**	**Q**	**∧**	**NAND**	**∨**	**∨_*e*_**	**NOR**
T	T	T	F	T	F	F
T	F	F	T	T	T	F
F	T	F	T	T	T	F
F	F	F	T	F	F	T

Columns 1–2 show propositions P and Q and the different combinations of the truth values logicians assign to them in terms of whether the propositions describe a state of the world or not (T, true; F, false). The remaining columns specify the truth tables for the logical connectives Conjunction and NAND (columns 3–4), Inclusive and Exclusive Disjunctions (5–6), and NOR (7).

On the side of non-linguistic, reasoning-related properties of compound sentences, the interpretation of a disjunctive sentence such as *the triangle is yellow or the square is blue* can sometimes raise the question of whether only one of the clauses is in fact true, thus requiring that the other clause be discarded (Mascarenhas and Koralus, 2015). This interpretation usually surfaces when a disjunctive sentence is presented as the premise to a problem-solving task; in such circumstances, participants appear to apply a “disjunction elimination” strategy, as in formal logic. This rule of inference determines that, when presented with premises *P or Q* and *Not P*, it follows that *Q* must apply (or be true); this particular inference rule has been investigated in adults as well as in both verbal and preverbal children (Mody and Carey, 2016; Cesana-Arlotti et al., 2018), and the results indicate that both infant and adult participants conform to it.

Inference rules of this sort are plausibly the result of post-linguistic processes, given that they tend to materialise when participants are asked to reason with compound sentences. From the perspective of language processing, in fact, inference rules need not apply at all; a disjunctive sentence may potentially apply to three different states of affairs, in accordance to its truth table, but in regular linguistic exchanges the comprehension system regularly computes a single interpretation only and with no need for any reasoning strategy to do so (such rules are not automatic or mandatory in any way). The overall context as well as the nature of linguistic exchanges help hearers establish the intended interpretation, quickly so and with little, if any, reflection.

Having said that, the relationship between the linguistic and non-linguistic properties of compound sentences is necessarily a subtle affair, not least because in order to reason with these sentences adequately they must be comprehended appropriately. That is, the language comprehension system must be able to access all possible readings of compound sentences when required to do so if whatever mental systems are in charge of reasoning are to successfully apply each reading of a compound sentence as premises to an inference rule. In such cases, the mapping between the linguistic system and the relevant reasoning system can be rather blurry, though the respective processes certainly differ in important ways. In particular, language comprehension is mostly an implicit process not available to introspection, while the ability to represent all possible interpretations of compound sentences, and indeed to reason with such readings, is more explicit in nature. After all, it is clearly possible to reflexively consider what such sentences mean as well as what may follow from them, even if the actual details of how these interpretations and consequences are obtained are not directly accessible.^2^

The overall picture illustrates the fact that there are different facets to the interface between the processes involved in the comprehension of language's logical connectives and the processes at play in the representation of all possible readings of compound sentences, and these aspects need to be carefully considered in an experimental investigation of these issues (a study of the language-and-thought interface, in effect). With this purpose in mind, we put together a tailor-made experiment to probe some of the implicit processes underlying the (fast) comprehension of compound sentences as well as some of the more explicit (and slower) phenomenon of reflecting upon the different interpretations compound sentences yield. In particular, we combined the visual-world paradigm with a sentence-picture matching task, employed a seldom used but potentially fruitful statistical model to analyse eye-tracking data, and exploited the peculiar way in which the logical connectives are realised and used in language—the necessary armamentarium to track how participants represent the meanings of logical connectives.

The last point is central to the framework we implemented. It is noteworthy that even though there are a possible 16 binary connectives in logic, and language makes use of a great number of coordinators to link up different clauses (*and, because, if…then*, etc.), only two coordinators behave like logic's connectives. That is, of the 16 possible binary logical connectives, only two have been unambiguously lexicalised in the world's languages: the aforementioned conjunction *and* and (inclusive) disjunction *or* (Horn, 2012), resulting in the relevant types of logical compound sentences, conjunctive and disjunctive sentences (we put to one side the issue of cross-linguistic variability in order to simplify matters, but in any case our discussion is uncontroversially true of the language of the experiments).

This is not to say that natural languages lack the resources to express or describe the possible state of affairs that the 16 logical connectives can account for; indeed, the truth tables of non-verbalised connectives can be derived analytically through the combination of *and, or*, and negation, a fact of language as much as of formal logic (Guttenplan, 1997). In fact, all 16 truth tables can in fact be derived by the repeated application of one single operator from logic, either the alternative denial connective, also known as NAND or Sheffer's stroke (column 4 in Table 1), or the joint denial connective, often referred to as logical NOR (column 7). In either case, the representation of certain meanings would require significantly long strings of derivations and as a result quite convoluted sentences, resulting in a rather inefficient medium of communication, which may partly explain why these two connectives have not been lexicalised in any language (the English word *nor*, which is mostly usually within the correlative connective *neither/nor* or under negation, should not be confused with logical NOR, though there might be some connection between the two; Horn, 1989).^3^

There has been plenty of discussion in the literature as to why natural languages have verbalised a specific set of connectives and not another; or said otherwise, why some connectives have been blocked from being lexicalised. Most explanations have pointed to so-called economy considerations regarding the derivability of the non-verbalised connectives, in addition to a restriction on “negative” connectives—NAND and NOR are examples of such connectives, as the former is the contrary, or opposite, of conjunction *and* and the latter of inclusive disjunction *or*, as can be ascertained in Table 1 by contrasting columns 3 and 4, on the one hand, and columns 5 and 7, on the other (the truth tables are reversed, as it were; Gazdar and Pullum, 1976; Horn, 2012; Katzir and Singh, 2013). Crucially, these “blocking effects” do not actually establish that the unlexicalised connectives constitute so-called “impossible words” (Collins, 2011); they just attempt to explain why some connectives and not others have been lexicalised.

Some concepts do appear to be genuinely impossible to lexicalise—i.e., to become established words of a language—perhaps for metaphysical reasons (Fodor and Lepore, 2002) or on account of intrinsic linguistic constraints (Collins, 2011), but the concepts themselves seem perfectly entertainable in a conceptual representational system of the mind, a *language of thought* (Fodor, 1975). Natural languages clearly confer a great deal of flexibility when it comes to inventing new words, especially in the case of so-called open class words such as nouns and verbs, though it is noteworthy that some verb forms appear to be barred. Cinque (2013) offers the following sample of non-existent verbs in English (the verbs appear in italics, and the intended meaning within parentheses):

(1) He has *climbend* the tree [“he has worryingly (for the speaker) climbed the tree”]
(2) He *fightaf/runaf* (“he is afraid of fighting/running”)
(3) He *didish* it (“he did it shamelessly”)
(4) I *sayam* you are wrong (“I am sympathetic in saying you are wrong”)

The meanings these verbs convey are not hard to grasp, for the corresponding concepts are perfectly entertainable (they can be mentally represented); it is just that these concepts cannot be lexicalised in this particular way. As mentioned, there are a number of proposals on offer as to why this is the case; Fodor and Lepore (2002) had it that verbs such as these typically refer to events that are in principle independent of each other—*worrying* and *climbing a tree, being afraid* and *running*, etc.—and this would go some way toward explaining why such verbs are disfavoured, whilst Collins (2011) has argued that lexical items exhibit a complex internal structure (they are not semantically simple) and this very structure often precludes some intended interpretations. Be that as it may, and whatever the details, the consensus is that such verb forms are indeed not possible.

More importantly, these data yield important information on the sort of primitives thought allows for, including the sort of things we can entertain and think about without the need, and in some cases without the possibility, to employ linguistic vehicles. Or in the words of Cinque (2013), what seems to be happening here is that “of all the concepts and distinctions that populate our system of thought only a fragment receives a grammatical encoding in the languages of the world” (p. 50). This claim is not unrelated to the often-made point that the connection between linguistic and conceptual representations will rarely ever be, strictly speaking, one-to-one—say, one thought per sentence, one meaning per word, etc. (Pickel, 2019; makes this point, though in a slightly different context).

This general state of affairs—the split between learnability and lexicalisation, on the one hand, and the mismatch between linguistic and conceptual representations, on the other—is applicable to the case of unlexicalised logical connectives too, the very topic we focused on in this study, with the further proviso that coordinators and connectives are so-called *function* words, or closed class words, and do not usually yield new coinages, as opposed to open class words like nouns and verbs, which do allow new members, as noted.

There has been some discussion in the literature as to why so few connectives have been lexicalised, but little about the possibility that non-lexicalised connectives may be learned in an appropriately designed experiment—and yet this suggests a potentially valuable way to explore non-verbal properties of cognition (Piantadosi et al., 2016 is an exception, though their take is rather different from ours here). The key point is that a theory of what makes lexicalisation possible in some cases but not in others does not preclude the eventuality that unlexicalised connectives may be learned; that is, if there are no reasons to believe that unlexicalised connectives constitute impossible words, then it ought to be possible to devise an experimental task in which participants would be expected to learn and appropriately comprehend made-up words standing for unlexicalised connectives. In other words, if a concept is entertainable and it *could* have been lexicalised had the facts of the matter been conducive to it, then a word meant to embody the meaning of such a concept could be learned in an experimental setting. A theory of the lexicalisation (or lack thereof) of the connectives is not *ipso facto* an account of the learnability (or not) of non-existent words, pointing to a theoretical split between lexicalisation and learnability.

The literature contains a couple of examples of how the split can be empirically tested. Hunter et al. (2011), in particular, report a study on the learnability of unlexicalised determiners—determiners that do not exist (it is worth noting that determiners are also closed class words). The concept under analysis in this study was the unattested determiner *fost*, which would be the opposite of the existing determiner *most*, thereby meaning “less than half” (*most* clearly means “more than half”). Interestingly, *fost* could in fact exist, as it exhibits the same semantic properties as *most*—both determiners are what semanticists call “conservative”—and there are no other internal or metaphysical reasons barring its lexicalisation (it seems that this determiner does not exist for pragmatic reasons). Since *fost* could in fact exist, it ought to be possible to design an experiment in which participants were required to learn how to use this non-existent word. According to the data Hunter et al. obtained, both young children and adults can indeed learn and appropriately use the meaning of *fost* when put to the test. These data suggest that certain unlexicalised concepts are indeed entertainable, as it is reasonable to suppose that the meanings of unattested words would only be learnable if the corresponding concepts are in fact available in the *language of thought*.

This specific issue is not actually new. Fodor et al. (1974) entertained a similar problematic some forty or so years ago, the interest then lying on the issue of whether terms that are simple in language are connected to complex representations in thought. Among other data, they discussed the “simple” word *below*, which they argued was connected to the conceptual representation NOT ABOVE (I capitalise concepts, as per the norm), thus claiming that *above* would correspond to a primitive in the *language of thought*, namely ABOVE, while the word *below* would correspond to a complex representation, the combination of the concepts NOT and ABOVE. That is, the concept for the word *below* would be constructed from the concepts NOT and ABOVE rather that there being an independent BELOW concept. It is fair to say that subsequent results were equivocal, and Fodor at least moved on from such arguments in subsequent publications of his. Still, the logic of the approach is not faulty, as the Hunter et al. study demonstrates, and non-existent logical connectives constitute a rather apt case of other possible words that do not exist but could have had the facts of the matter been different.

Non-existent linguistic connectives can certainly be entertained in thought—they *have* been entertained, in fact, and explicitly so, by logicians and philosophers—but they have not been lexicalised in language, in any language, *qua* natural language connectives—that is, words such as “nand” and “nor” do exist, at least in English, but they are technical terms within the field of formal logic, theoretical constructs that play a specific function within their field, but they are not actual linguistic coordinators that the languages uses to put two clauses together. So framed, the conceptualisation of the logical connectives appears to be divorced from their lexicalisation, and thus are ripe for experimentation. The unlexicalised connectives NAND and NOR are good candidates for such an undertaking, given their connection to the existing linguistic connectives *and* and *or*, and the fact, derived from this tie, that they do not express especially convoluted state of affairs—e.g., neither is only true, for example, in case the first clause of a compound sentence is true but false in any other instance, which would be an odd linguistic structure indeed (such a meaning corresponds to the truth table of the contrary of *material implication*, actually, naturally an unlexicalised connective).

In this study, we specifically targeted the NAND connective, as its truth table specifies three possible situations, for just one for NOR, and thus the learning of this unlexicalised connective would be a clearer demonstration that the requisite concept is not only entertainable but that it can, furthermore, be appropriately applied to a non-existent word. Indeed, in comparing the difficulty of learning new words for NAND and NOR, the latter might be too easy a task, as a strategy that simply negates the two clauses of the presented sentences would suffice to show competence with the meaning of the made-up word for NOR (recall the truth table of NOR in Table 1, with its single true value). After all, in order to demonstrate that the NAND concept is available, and moreover, applicable to a novel word, participants would have to accept the truth of a NAND compound sentence when either one of the two clauses is true (and the other false), *in addition* to when both clauses are false, and this would necessitate a more nuanced interpretation than in the case of NOR.

Having said that, it is important to stress that the connectives NAND and NOR form a superset/subset relationship, as the truth table of NOR is a subset of the truth table of NAND. Indeed, NAND and NOR sentences are both true when the two clauses are false—an FF reading—and this could introduce some ambiguity in an experiment, as a made-up word for NAND could easily be applied to the NOR concept instead, even within a well-designed training session. This is because compound sentences typically receive what may be termed default readings in psycholinguistic experiments (Lobina et al., 2022), much like ambiguous sentences in general do, and it was reasonable to expect that the FF reading would turn out to be the participants' preferred reading for NAND sentences. After all, the NAND connective is the contrary of *and* and an FF interpretation is a straightforward negation of the TT reading, the only true value of conjunctive sentences. We kept this issue firmly in mind when designing and running the experiments, as we discuss below. We now turn to the details of the experiments we ran.

## 2. Materials and methods

**Figure 1:**
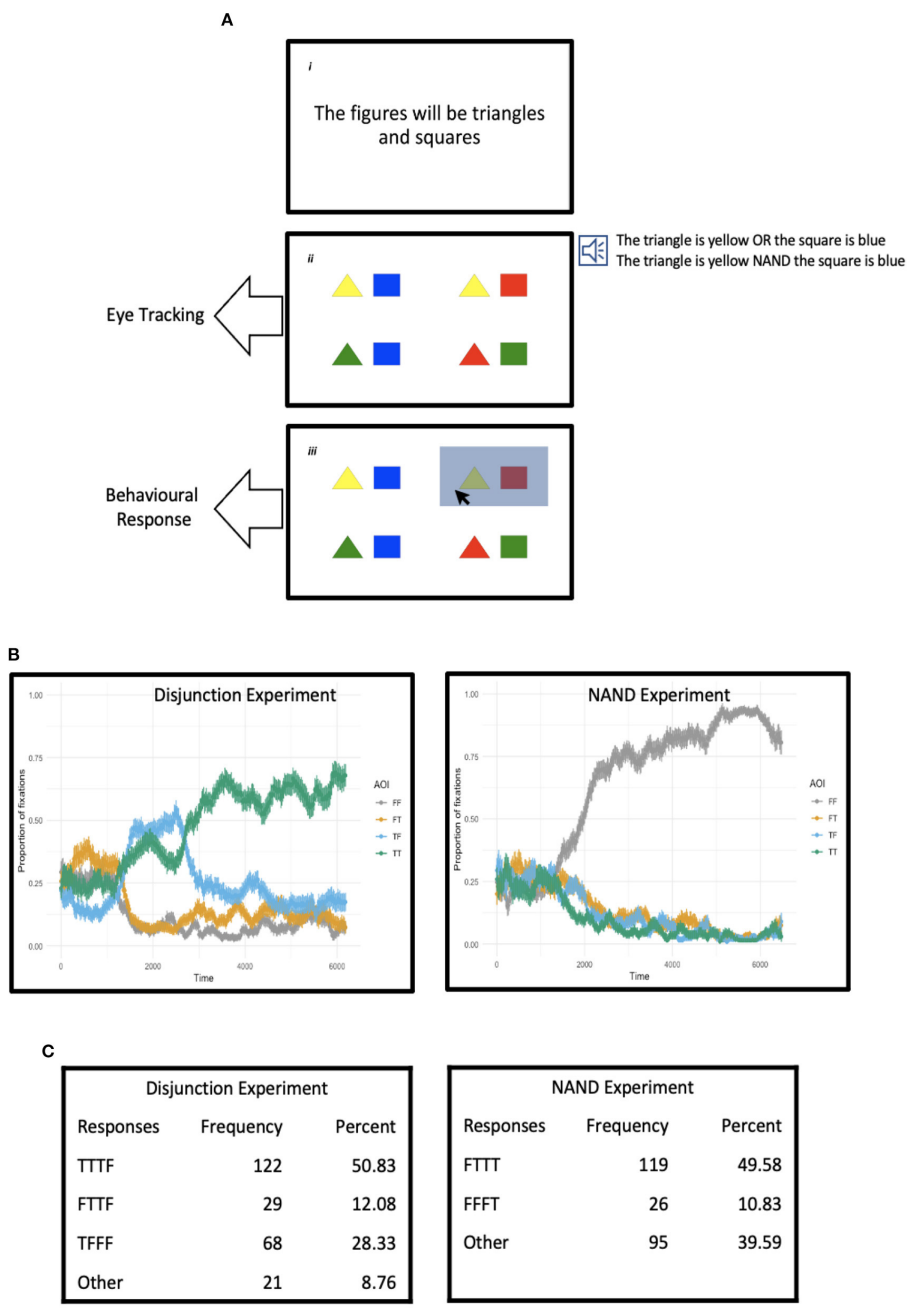
Interpreting and thinking with existent and non-existent verbal connectives. **(A)** (i) Panel specifies the figures participants see in a trial. (ii) Each quadrant in the experimental panel presents two figures in diverse combinations of colours. An audio of a sentence describing one or more quadrants is played soon after the figures appear, as shown to the right of the panel. Each quadrant represents a combination of the truth values of two propositions (namely, the combinations TT, TF, FT, and FF), while the sentence played to participants is a linguistic representation of two propositions mediated by a logical connective. The tracking of eye movements starts from the beginning of the sentence and continues for a further 3 s after the sentence finishes. (iii) At the end of the eye-tracking, the mouse pointer is activated and participants are asked to select all the quadrants that match the sentence. **(B)** Proportion of fixations to each quadrant (TT, TF, FT, and FF) for a duration of circa 6,500 ms (3,500 ms for the longest sentence plus 3,000 ms of “looking time”). **(C)** Behavioural responses, where the TTTF pattern, for instance, specifies that participants had selected quadrants TT, TF, and FT.

We employed the visual-world paradigm in combination with a behavioural sentence-picture matching task, as described in Figure 1A. The visual-world set-up was slightly different from what is usually the case in the field; a typical design uses four regions or areas of interest on a computer screen, each displaying a graphic, in order to use one graphic as the target interpretation for some aspect of the sentence played to participants, one graphic as a potential competitor, and two further graphics as distractors (Tanenhaus and Trueswell, 2006). In our set-up, the graphic on each quadrant was instead a representation of a possible combination of the two values from each line of a truth table—namely, the combinations TT, TF, FT, and FF (columns 1–2 from Table 1)—and thus which combinations would properly match the sentences presented to participants—i.e., which graphic would constitute a true statement of the graphics—would depend on which connective is used to put together the two clauses. In the case of conjunctive sentences, for instance, only the quadrant representing the TT combination would be the right interpretation for these sentences, while three such quadrants would match conjunction's opposite, the NAND connective (as mentioned, quadrants TF, FT, and FF).

We carried out two experiments; in addition to a task with NAND sentences, Experiment 2, the target of the study, we also conducted an experiment with disjunctive sentences, Experiment 1, wherein three quadrants were also applicable in this case too (namely, TT, TF, and FT). The overall study was thus divided into two main experiments, which were administered in the same session and in order, with a break between the two experiments; in the first part of the session, participants would carry out Experiment 1 with disjunctive sentences, as described in Figure 1A, whilst in the second part of the session, participants would undertake Experiment 2, which exhibited the same sort of design, but in which the task would be conducted with compound sentences where the two clauses would be mediated by a non-existent but possible word standing for NAND. The disjunction experiment was included to familiarise participants with the task so that there were no extraneous effects of any kind during the NAND experiment; disjunctive sentences, in addition, constituted a good control for both the analyses we intended to conduct and participants' performance with NAND sentences, as we discuss below. The NAND experiment was itself divided into two parts. Participants would start this session by undertaking a learning phase in which they would be shown a series of situations that NAND sentences appropriately describe (or not, as in the case of the TT reading, the one *false* interpretation for NAND sentences). Once the learning phase was completed, participants would carry out the same kind of task they had performed in the experiment with *or*.

As described in Figure 1A, the underlying idea of the study was to track participants' eye movements as the compound sentences are aurally presented, from the start of the audio file until a few seconds after the audio has finished, at which point participants were asked to select as many quadrants as they thought properly matched the sentence they had just heard. Such an experimental procedure would produce three relevant pieces of information. The eye-tracking data during the sentence is played, including when the sentence ends, would provide an online, implicit record of language comprehension processes. The participants' eye movements immediately after the sentence has finished and for a period of a few more seconds afterwards would plausibly coincide with the more explicit processes related to working out the various interpretations the compound sentences allow, if this is indeed the case. And finally, the forced choice responses at the end of the task would reflect the actual reflexive process of deciding which readings are definitely warranted. Put together, these data would provide a fuller picture of the comprehension and reasoning processes involved in entertaining and linguistically expressing complex mental propositions.

Regarding the hypothesis, and to start with the eye-movement data, participants were expected to naturally zero in on what might well be regarded as the default meanings of disjunctive and NAND sentences, notwithstanding the fluctuations that were likely to occur during each time-series, as the truth tables for disjunction and NAND each contain three true combinations. As myriad studies in psycholinguistics have determined, participants eventually put together a single interpretation for the sentences they are exposed to in an experiment, even when these sentences are ambiguous in various ways; indeed, very often these interpretations are regarded as the default readings of the sentences so employed, and this point is the more relevant here given that the experimental setting we employed was meant to mitigate pragmatic effects and thus focus on semantic properties only (we used the general design described in Lobina et al., 2022, which successfully neutralised such effects; we defer to that study for details).

Thus, in the case of Experiment 1, we expected participants to converge to the TT reading, following data from previous studies (Lobina et al., 2022), even if overall attention was anticipated to be divided between the TT reading and the mixed forms TF and FT during and immediately after the sentences were played, as per the expectation that the exclusive reading of disjunction is quite prominent in communication. As for Experiment 2, we hypothesised that the preferred reading would be the FF interpretation, possibly the default meaning of a connective that is effectively the opposite, or contrary, of *and*, though the mixed forms TF and FT would need to be considered too if NAND sentences are fully understood, and indeed, interpreted logically. Regarding the behavioural responses, we predicted that if participants were to demonstrate mastery of the full meaning of disjunctive and NAND sentences, the most common pattern of responses would be TT-TF-FT for disjunction *or* in Experiment 1, and TF-FT-FF for NAND in Experiment 2 (that is, patterns TTTF and FTTT, respectively), in accordance to their truth tables.

The overall set-up presented some analytical challenges in the case of the eye-tracking data, however. The quadrants participants would be exposed to, showcasing the TT, TF, FT, and FF combinations, were all potentially relevant and thus fixations on all four areas of interest needed to be tracked—and for a fairly long period of time in each trial to boot (roughly, 6 s), as we were interested in identifying when exactly during the time course of a trial the differences between quadrants would in fact surface. Such a long record of eye-movement data was also likely to give rise to a high amount of autocorrelation, a common occurrence in experiments dealing with time-series (Baayen et al., 2018). And finally, an initial inspection of the data suggested a non-linear pattern, as indicated by the wiggliness of the lines representing proportions of fixations shown in Figure 1B, generated prior to the analyses. Thus, a commonly-used technique to analyse eye-tracking data such as the linear regression implemented in a *growth curve analysis* (Mirman et al., 2008) was not adequate for the purposes of the study and other techniques used in the past are not methodologically sound (Barr, 2008).

We employed a *generalized additive mixed-model* (GAMM) (Wood, 2017) to analyse the eye-tracking data, a technique that is becoming more common in analyses of time-series data (Baayen et al., 2017, 2018) in cognitive psychology, including a few eye-tracking studies (Montero-Melis and Jaeger, 2019). GAMMs are specifically useful on account of three features: the models relax the assumption of a linear relationship between predicting variables and response variables by implementing so-called “smooth functions”; the autocorrelation of the data can be accounted for by the inclusion of an autoregressive model; and, particularly convenient for the aims of our study, the interpretation of these models is partly determined visually, allowing us to plot, among other things, the differences between conditions over time (see Supplementary material for further discussion of this method).

### 2.1. Experiment 1

#### 2.1.1. Participants

Fifteen psychology students (1 male, 14 female) from the Rovira i Virgili University (Tarragona, Spain) participated in this and the next experiment for course credit. None of the participants had undertaken any course in logic or reasoning before taking part in the experiments. All participants carried out Experiment 1 first, and after a short break, Experiment 2. The mean age was 18.5 years (*SD* = 0.68), and none of the participants had any known hearing or visual impairments. All were native speakers of Spanish. All participants gave their written informed consent before taking the experiment, the experiments followed the Rovira i Virgili University research and ethical guidelines, and the overall study itself was approved by the University's Ethics Committee on Research into People, Society, and the Environment (reference: CEIPSA-2021-PR-0024).

#### 2.1.2. Materials

This experiment evaluated a single condition with four levels. Each level corresponded to each of the four values of the inclusive disjunction's truth table (TTTF). Sixteen biclausal, declarative Spanish sentences, with the two clauses connected by the coordinator *or*, were constructed. Each clause ascribed a single colour to a single geometrical figure; we used four different colours (blue, yellow, red, and green) and four different figures (circles, squares, triangles, and diamonds). Sixteen more sentences were constructed to act as fillers. The fillers were monoclausal and thus only one figure was mentioned, but in this case the figure was ascribed two colours instead of one by employing the connective *and* and the overall sentence was furthermore negated (e.g., *the circle is not blue and green*). A further eight practice sentences were created, four of which were similar to the experimental items and four to the fillers. The average length of all sentences was 2,555 ms and the longest sentence was around 3,000 ms. The sentences were recorded in stereo with a normal but subdued intonation by a native, male speaker of the Spanish language using the Praat software on a Windows-operated computer. The graphics representing each one of the truth values of inclusive disjunction as well as the truth values of negated conjunctions (for the fillers) were created with Microsoft PowerPoint.

#### 2.1.3. Procedure

The experiment was designed and run with the Experimental Builder software (SR Research Ltd.) and administered in a laboratory with low to normal illumination in which each participant was tested individually. Participants were seated in front of a computer screen and were asked to place their head on a chin rest. The chin support was adjusted for each participant so that there was a distance of around 60 cm between their eyes and the monitor where the visual scene was presented, a 19-inch screen set to a resolution of 1,024 × 768 pixels. The position and fixations of participants' right eye, most people's dominant eye, were continuously recorded at a sampling rate of 1,000 Hz with an EyeLink 1000 eye tracker. In addition to the eye-tracking data, the participants' behavioural responses at the end of the trials were also recorded.

The overall flow of the experiment as well as the general design is shown in Figure 1A. Each trial started with a fixation point in the middle of a white screen. Participants were asked to fixate on this point and to press the space bar when they were ready to start the trial. A sentence such as *the figures will be triangles and squares* would replace the fixation point 500 ms after pressing the space bar. The sentence would stay on the screen for 2,500 ms so that participants had enough time to read it fully; at the end of this period of time, the sentence was replaced by the visual display, which would remain on the screen for the remainder of the trial. The display was divided into quadrants and a specific combination of figures and colours would appear on each quadrant, where the figures matched those mentioned in the sentence presented before the visual display (the placement of each graphic was randomised across quadrants and trials). After 2,000 ms, the time we allocated to participants to view the quadrants fully before presenting any other stimuli, a sentence describing one or more quadrants was played over headphones binaurally. Once the sentence had finished, there was a period of 3,000 ms of “looking time”, at the end of which the cursor would be activated so that participants could select the quadrants they thought the sentence described appropriately. The trial ended once participants were satisfied with their answers and had pressed the space bar to move on to the next trial (or reach the end of the session). Participants carried out an eight-item practice session with the experimenter, who explained the overall task and answered any questions before proceeding to the experimental session. Eye calibration was conducted before the practice session and again before the experimental session. The experimental session consisted of a total of 32 items, 16 of which were experimental and 16 fillers; the presentation of experimental and filler sentences was randomised. The experiment lasted 15 min overall.

#### 2.1.4. Data analysis

##### 2.1.4.1. Eye-tracking data

The eye gaze data collected with the EyeLink 1000 eye tracker was exported by using the manufacturer's Data Viewer software. The Sample Report this software outputs requires significant preprocessing before analysis and plotting, and we used the R package *VWPre*, version 1.2.4, for this purpose (Porretta et al., 2016). To begin with, we performed an analysis of trackloss (i.e., the amount of times the eye tracker lost track of participants' eye gaze); 1.68% of data was marked as off-screen and 4.86% as trackloss, and as a result 7 trials with <75% of data were eliminated (this threshold is common in the literature and seemed reasonable for our own experiments too). The data were then prepared in order to conduct a logistic GAMM analysis. As the task was effectively underlain by a four-way, multinomial choice and thus all quadrants were potentially valid interpretations for the sentences, the factor “area of interest” (AOI) was the main predictor in our models and constituted the indicator variable for the coding. The data were coded on a millisecond by millisecond basis, where a fixation on a given AOI at a given time was coded as a “1” (for success) and a non-fixation as a “0” (for failure).

Logistic GAMMs were conducted and partly analysed with the R package *mgcv*, version 1.8-35, family class binomial (Wood, 2017). Three such models were run and compared, and their analysis was complemented with the R package *itsadug*, version 2.4 (van Rij et al., 2020). We used model selection to determine the best random-effects structure for the data, which is the most appropriate approach for non-linear models such as GAMMs (Baayen et al., 2017; Wieling, 2018).

An important factor to consider when using the *mgcv* package is that fitting GAMMs often requires significant computational resources and some processes may take a very long time to complete, in some cases even days (fitting such models certainly takes much longer than fitting linear mixed-effects models with the *lme4* package, the most commonly used package in cognitive psychology). Given that in the 6,000 ms of eye movements we recorded in each trial a pattern was pretty evident after 4,000 ms and did not change in any way after that, as shown in Figure 1B for both experiments, we decided to reduce the length of the time-series to analyse to 4,000 ms, which we hoped would ease the demands of the analyses. The average length of the experimental sentences from Experiment 1 was 2,947 ms and this meant that the models we analysed included at least 1,000 ms of “looking time,” which was abundantly sufficient for the purposes at hand and no important effects were expected to be missed. Full details of the models we ran as well as the model comparison procedure we followed in this and the next experiment can be found in the Supplementary material file. In addition, the overall data as well as the R script for the analysis of the eye-tracking data of both experiments are available at: https://osf.io/mfqt8/?view_only=dc416b5ac605423b80449714fd0f4979.

##### 2.1.4.2. Behavioural responses

For the analysis of these data we drew a distinction between acceptable (or correct) patterns of response and unacceptable (or incorrect) patterns in order to run chi-squared tests between the expected and observed responses, in two steps: first between acceptable and unacceptable responses as a way to confirm that the sentences had been interpreted appropriately, and then within acceptable responses between the two patterns of interpretation we had identified beforehand as being applicable in each experiment.

### 2.2. Experiment 2

#### 2.2.1. Participants

The same as in Experiment 1, as noted above.

#### 2.2.2. Materials

This experiment also evaluated a single experimental condition with four levels, but in this case the two clauses of the compound sentences were connected by a non-existent but possible word in Spanish standing for the unlexicalised logical connective NAND (truth table: FTTT): *fro*. The nonsense word *fro* is not related to, nor does it resemble, any other word in Spanish (or Catalan; the experiment took place in Catalonia), it is easy to pronounce, and its morphology favours the sort of use participants would be exposed to in the experiment (i.e., as a coordinator). We used the same figures and colours employed in Experiment 1, and a total of 32 NAND sentences were constructed, 16 experimental sentences and 16 sentences meant for the learning phase. As for the filler sentences, these were also similar to those of Experiment 1, but in this case the monoclausal sentences were not negated. A further eight practice sentences were created, four of which were similar to the experimental items and four to the fillers. The average length of all sentences was 2,837 ms and the longest sentence was around 3,500 ms. All other details remained the same as in Experiment 1, except that the graphics for the experimental sentences now represented each one of the values of the truth table for NAND and the graphics for the fillers the values of the truth table for (non-negated) conjunctive sentences.

#### 2.2.3. Procedure

The procedure was very similar to that of Experiment 1, with the addition of a learning phase for the connective NAND. In this phase, which was undertaken before the practice session, participants would be shown a series of situations that NAND sentences could appropriately describe (or not). In particular, participants would be exposed to individual graphics in each trial, with each graphic always depicting two geometrical figures in two different colours, as in the experimental materials. The actual procedure of the learning phase was as follows: the graphic would appear on the screen first, and after a brief period of time allocated to participants so that they could inspect it adequately, a NAND sentence would be played over the headphones. Soon after, participants would be presented with a feedback on screen indicating whether the sentence was an appropriate description of the graphic or not—with a tick, for “yes,” and an X for “no.” Participants undertook four iterations of the truth table for NAND, for a total of 16 trials; that is, the four values of the truth table of NAND—viz., FTTT—were repeated four times so that participants were exposed to 16 different NAND sentences (the order of presentation was randomised). There was no explicit instruction of any kind, nor did participants have to complete any task; instead, the learning phase was similar to what is employed in artificial grammar learning studies (Fitch and Friederici, 2012), a learning by exposure scenario, and the expectation was that participants would implicitly learn the meaning of the novel word being presented to them given a specific set of scenes (a similar strategy is usually employed in studies of language acquisition, though in this case studies concentrate on content words such as nouns and verbs; see, for instance, Akhtar and Tomasello, 1997). Once this phase had been completed, participants undertook an 8-item practice session and right after an experimental session of 32 items (16 experimental sentences, 16 filler sentences); all other aspects of the experiment remained the same as in Experiment 1 (4 areas of interest per trial, randomization, etc.). Eye calibration was conducted before the practice session and again before the experimental session, but not before the learning phase. This experiment lasted around 20 min overall.

#### 2.2.4. Data analysis

##### 2.2.4.1. Eye-tracking data

The eye gaze data was prepared in the same way as the data from Experiment 1 was prepared. In this case, an analysis of trackloss marked 1.83% of data as off-screen and 6.08% as actual trackloss, and thus 11 trials with <75% of data were eliminated. In this experiment too the time series were reduced to 4,000 ms and this yielded around 640 ms of looking time (the average length of experimental sentences was 3,366 ms), which was also amply sufficient for the analyses, as the overall pattern of this experiment was in fact established significantly early, around 1,400 ms. GAMMs were processed and analysed in the same way as in Experiment 1.

#### 2.2.5. Behavioural responses

These data were analysed in the same way as the data from Experiment 1.

## 3. Results

The overall data are shown in Figures 1B,C. According to the eye-movement record, participants eventually converged on the TT interpretation for *or* in Experiment 1, though the mixed form TF also received a significant number of fixations, while for NAND in Experiment 2 participants preferred the FF reading from very early on and did not divert from this interpretation. At first sight, this would suggest a preference for a conjunctive interpretation of disjunction and a NOR, FF reading for the NAND connective.

**Figure 2:**
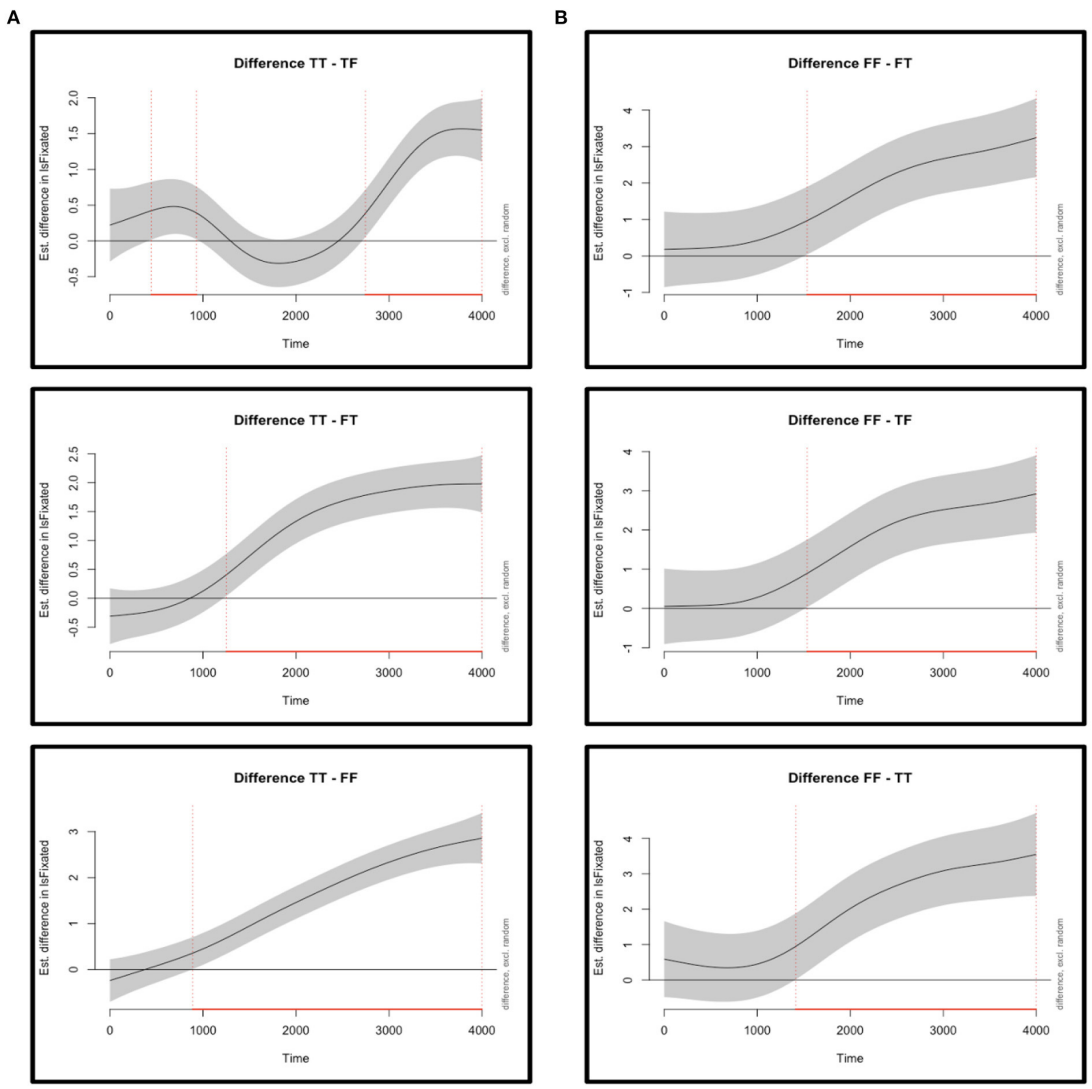
Difference curves, derived from the best-fit model of a generalized additive mixed-model analysis. The graphs show the comparison between the (non-linear) smooth of the quadrant with the most fixations (fixations are labeled as IsFixated on the y-axis) against each of the (non-linear) smooths of the other quadrants, with the gray solid line indicating the estimated difference. The shaded band represents the pointwise 95%-confidence interval; when the band doesn't overlap with the x-axis (i.e., the value is significantly different from zero), this is indicated by a red solid line on the x-axis along with red vertical dotted lines. The graphs show fixations during the audio of the entire sentence in addition to an extra 640–1,000 ms of “looking time” (see Supplementary material for the selection of this time-window for the analyses). **(A)** Estimated differences from Experiment 1 between fixations to the TT quadrant and fixations to the TF, FT, and FF quadrants. The TT-TF comparison exhibits differences in two time windows, at window 445–930 ms, roughly around the time the first clause is being played in the audio, and at 2,750–3,999 ms, where the beginning of this time-window coincides with the end of the audio. The TT-FT contrast produces a difference at 1,250–3,999 ms, a window that (roughly) starts right after the connective has been presented, while the TT-FF comparison exhibits a difference at 890–3,999 ms, where the beginning of this time-window precedes the presentation of the connective. **(B)** Estimated differences in Experiment 2 between fixations to the FF quadrant and fixations to the FT, TF, and TT quadrants. Comparisons show that all differences surfaced after around 1,400 ms, when the connective has already appeared and the second clause is being presented.

In the case of Experiment 1, the best-fit GAMM included smooth functions for time per condition (TT, TF, FT, and FF), the fixed effect, as well as smooths of time per time-series (i.e., per trial) per condition (TT, TF, FT, and FF), accounting for the random effects, and in every case non-linear curves were obtained, as confirmed by the effective degrees of freedom (edf), a summary statistic of GAMMs that reflects the degree of non-linearity of a curve. An edf equal to 1 corresponds to a linear relationship between the predicting variables and the response variable, and anything above 2 equals to a highly non-linear relationship, which is what was observed in this model for every one of the eight smooth functions (*ps* < 0.001). Figure 2A shows the “difference curves” between the smooth function for TT, the most fixated quadrant, against the remaining three smooths (for TF, FT, FF). Difference curves are the appropriate way to evaluate differences among the various levels of an experimental condition in GAMMs, in this case the main fixed effect of “area of interest” (or quadrant), showcasing the importance of the visual inspection of the data in these models. Indeed, these curves allow researchers to plot when exactly during trials the estimated differences actually arise, and Figure 2 both marks and describes the relevant time windows for each comparison in each experiment (note the relative variability from Experiment 1, where the TT-TF comparison is the most important one).

In the case of Experiment 2, the best-fit GAMM included smooth functions for time per condition, the fixed effects, as well as by-participants and by-items smooth functions for time per condition, the random effects, for a total of 12 curves. Here too the non-linearity of all curves was confirmed, as the edf value was above 2 in every case (again, with *ps* < 0.001). Regarding the difference curves between the smooth for FF, the most fixated quadrant, against the smooths for FT, TF, and TT, and as shown in Figure 2B, participants settled on the FF interpretation soon after the connective appeared and they kept their fixations on that quadrant for the remainder of the trial (the Supplementary material provides the full details of the analyses of both experiments, including the extensive summaries of the statistics).

Regarding behavioural responses, shown in Figure 1C, the preferred pattern of response was TTTF for disjunction *or* (that is, participants selected the TT, TF, and FT quadrants), and FTTT for NAND (participants selected TF, FT, and FF), in line with the respective truth tables. For the analysis of these data, and as mentioned above, we drew a distinction between acceptable patterns of response and unacceptable (or incorrect) patterns in order to run chi-squared tests between the expected and observed responses, in two steps: first between acceptable and unacceptable responses as a way to confirm that the sentences had been interpreted appropriately, and then within acceptable responses between the two acceptable patterns of interpretation we had identified for both disjunction and NAND.

In the case of disjunction *or*, Experiment 1, we took the patterns TTTF and FTTF to be the only applicable responses, as these are the truth tables of inclusive and exclusive disjunction, respectively, and everything else was regarded as a possible mistake in interpretation. In this case too exclusive and inclusive disjunctions form a superset/subset relationship, as language's *or* is indeed often ambiguous between the two truth tables—in our psycholinguistic parlance, the ambiguity is between two readings/interpretations: one that accepts a disjunctive statement when the state of affairs is TT, and one that does not. The percentage of applicable answers thus amounted to 67.41, for 32.59 of incorrect answers, and the difference between these two frequencies was significant [χ^2^(1) = 27.16, *p* < 0.001]. Once it was ascertained that the task had been appropriately carried out by the participants—the proportion of acceptable answers was greater than the proportion of incorrect answers—we compared the two patterns of acceptable responses. In this case, the percentage of responses for the inclusive interpretation of disjunction (TTTF) was 80.79, for 19.21 for the exclusive interpretation (FTTF), and this difference was clearly significant as well [χ^2^(1) = 57.29, *p* < 0.001].

The situation was slightly more nuanced for NAND, Experiment 2, as we had anticipated. Whilst the full interpretation of the NAND connective would correspond to the FTTT pattern, the FFFT response (that is, only quadrant FF is selected), properly speaking the truth table of the (also unlexicalised) connective NOR, cannot be regarded as an inapplicable or even an entirely incorrect response, given that these two connectives, as stressed, form a superset/subset pair—the truth table of NOR is a subset of the truth table of NAND—and this creates a possible lexical ambiguity in the made-up word we used in the training session. After all, the one true value that NAND and NOR share is the FF-only interpretation and this is the most straightforward reading for NAND as a counterpart to the TT reading of conjunction (recall that NAND is the opposite, or contrary, of conjunction). The eye-movement data do in fact indicate that the preferred interpretation for these compound sentences was the FF reading, and this further justifies the inclusion of FFFT as the second possible, acceptable response to the NAND condition, along with FTTT. Thus established, the overall percentage of applicable responses in this case was a total of 64.73, for 35.27 of unacceptable answers, and the difference between the two proved to be significant [χ^2^(1) = 19.45, *p* < 0.001]. And within acceptable or applicable answers, 82.07% of responses corresponded to the FTTT pattern and 17.93% to the FFFT response, and the difference was significant here too [χ^2^(1) = 59.65, *p* < 0.001], thus clearly favouring the full truth table for NAND.

## 4. Discussion

The results are noteworthy for a number of reasons. Firstly, the methodology and analyses we employed allowed us to probe the semantics of language's logical connectives in a more direct manner than has been the case before. Our approach was not centred on analysing the responses of a forced choice task (Chevallier et al., 2008; Lobina et al., 2022), nor did it involve evaluating which predictors are significant at specific (and short) time windows of an eye-movement record (Zhan, 2018). Instead, we tracked the full time-course of participants' eye movements as they processed compound sentences against a background of four potentially relevant scenes by using a GAMM to analyse these data, thereby establishing *when* exactly differences among the four readings arise. This was specifically done in order to contrast the (implicit) record of the cognitive processes involved in comprehending the sentences with the (more explicit) responses at the end of the trials, the latter the result of having to think through the sentences' full (logical) interpretation—that is, we carried out a methodology that contrasted the online measure of tracking eye movements with the offline measure of a sentence-picture matching task.

Thus set up, we were able to ascertain that participants settle on the primary interpretation of compound sentences early on, and moreover, that they do not diverge from this interpretation during the processing of a sentence, including during the few seconds after a sentence has ended, even if the scenes on display provide compatible interpretations and more reflexive processes as to what the sentences might mean could have been elicited at that point in such conditions. As mentioned, reasoning-related processes need not apply during the actual, online processing of a sentence, but when participants *are* asked to evaluate the overall meaning of compound sentences, as they were at the end of each trial in this experiment, they *do* demonstrate mastery of the relevant truth tables—an ability that is certainly part of what reasoning with compound sentences necessitates (and part, moreover, of the interface between comprehension and reasoning).

More to the (central) point of the study, this framework offers a compelling way to demonstrate whether participants are able to, not only conceptualise unverbalised connectives, but more importantly, match these concepts to novel words to boot. In so doing, we were aiming to substantiate the split between the lexicalisation of the logical connectives and the learnability of single, non-existent words meant to embody the meaning of unlexicalised connectives. Participants showed that they could, indeed, learn the NAND connective, and moreover, that they interpret the non-existent NAND sentences used in Experiment 2 in the same way that they interpret existent compound sentences such as the disjunctive sentences we employed in Experiment 1. That is, there was a clear preference for what may be regarded as the default meaning of NAND sentences in the eye-movement record, more so, in fact, than in the case of disjunction, though the other, valid interpretations of NAND sentences were also available when these were required during the sentence-picture matching task, much as with (inclusive) disjunctive sentences in similar conditions.

This result is of course directly related to the old issue of how language and thought relate, a question as unsettled as any other in the study of cognition. Putting to one side some of the issues the field has been concerned with in relation to this topic—for instance, whether certain thoughts necessitate a specific natural language—our evidence illustrates the long-held claim that linguistic representations do not exhaust what may be in general thought. This point can be traced back to medieval philosophy (and even earlier, as Panaccio, 2017 has chronicled), but the idea has only received a more rigorous characterisation in modern times. Apart from Fodor's *language of thought* hypothesis, already referenced earlier on, two “principles” are particularly relevant in the context of our study. One is due to Searle (1969), who argued in favour of a *principle of expressibility*, according to which whatever can be meant, or thought, can be said (p. 16), drawing a connection between what can be entertained in conceptual representations and what can be said in language. This principle is similar in kind to what Katz (1978) has called *effability*, the second principle we wish to underline: the typical inter-translatibility of whatever content one might be able to entertain, which is based on the apparent fact that whatever thought can be expressed in one language, it can also be expressed in another language in one way or another.^4^ Putting these two principles together, it would appear that we must allow for greater flexibility at the side of *what can be said* than at the side of *what can be meant/thought*. Or conversely, that the universe of the things we can entertain and think about appears to be greater than the universe of the things we can verbalise and talk about. After all, there are many different ways in which one may wish to linguistically communicate the very same thought, and these different ways will not always reflect conceptual differences.

It is certainly curious that we are capable of employing the resources of language to make some non-linguistic thinking processes available, or at least entertainable, as in the possibility of learning the meaning of made-up words standing for unlexicalised concepts such as the NAND connective, meanings that are not part of any natural language word at all, but we take it that this is in line with the equally long-held idea that what goes unsaid is not necessarily unthought, and that much of thought is probably unverbalised to begin with Fodor (1991). This, for us, suggests that the relationship between the capacity for natural language and the *language of thought* is more intricate than it is usually taken to be (Lobina, 2019), requiring a particular experimental perspective. The present framework should bode well for research on this very issue, as it ought to be possible to apply this approach to the study of other unverbalised properties of language, thereby tracking a fuller array of what can be humanly thought.

An appropriate development of our approach finds a natural home in the field of language acquisition, and the logical connectives have indeed received significant attention in this literature, though the experimental techniques so far employed have not been fit to test children below the age of 3 or 4 (Crain, 2012). This part of the field has consequently had little to say about the representation of the logical connectives in non-linguistic cognition, and yet the cognition of preverbal children—namely, children below the age of 3 or 4, often termed the “terrible twos”—could shed some light on the relationship between language and thought. The *visual world* paradigm, which when applied to young children has often been called the *intermodal preference looking paradigm*, basically a simplified version adapted to infants, should come handy for such an undertaking, as this technique has been successfully employed to unearth the linguistic knowledge and general cognition of children as young as 13–15 month-old, and more fruitfully so in the case of 18-month-old (Golinkoff et al., 2013). Thus, there would appear to be a wealth of data to be unearthed in this age group, and the *visual world* technique, in addition to a GAM analysis of eye movement data and our own experimental design, yields a methodology that is particularly apt for these purposes.

As a case in point, take the study described in Cesana-Arlotti et al. (2018), which was able to show that 12- and 19-month-old appear to demonstrate the ability to use the disjunctive syllogism by analysing infants' eye gaze data in the context of carefully controlled scenarios (the syllogism was termed a disjunction elimination strategy at the beginning of this paper). The ability to use the disjunctive syllogism requires also the ability to represent the concept of disjunction OR, and in the absence of language's disjunction to boot, as these children are yet to acquire the word *or*. What this study did not set out to do, however, was working out whether the meaning of the concept OR was inclusive or exclusive in these children's mental representations, an issue that was not all that relevant to the study, in fact, considering that the syllogism works just as well with either kind. And yet this would appear to be an important datum about how the *language of thought* develops in ontogeny, as the ability to use the syllogism, as noted, is seemingly available prior to the acquisition of the word *or*. We shall report on this issue anon.

## Data availability statement

The datasets presented in this study can be found in online repositories. The names of the repository/repositories and accession number(s) can be found at: https://osf.io/mfqt8/?view_only=dc416b5ac605423b80449714fd0f4979.

## Ethics statement

The studies involving human participants were reviewed and approved by Ethics Committee on Research into People, Society, and Environment, Universitat Rovira i Virgili, Tarragona, Spain. The participants provided their written informed consent to participate in this study.

## Author contributions

DL conceived the overall research. DL, JD, and JG-A designed the experiments. MG set up the experiments and supervised their implementation. DL and MG analysed the data. DL wrote the paper and JD, JG-A, and MG revised it. All authors contributed to the article and approved the submitted version.

## Funding

JD received funding from the Ministerio de Ciencia, Innovación y Universidades, from Spain (reference: PGC2018-094198-B-I00). The funders had no role in study design, data collection and analysis, decision to publish, or preparation of the manuscript.

## Conflict of interest

The authors declare that the research was conducted in the absence of any commercial or financial relationships that could be construed as a potential conflict of interest.

## Publisher's note

All claims expressed in this article are solely those of the authors and do not necessarily represent those of their affiliated organisations, or those of the publisher, the editors and the reviewers. Any product that may be evaluated in this article, or claim that may be made by its manufacturer, is not guaranteed or endorsed by the publisher.

## Footnotes

^1^We will often talk of a connective's (or more accurately, a compound sentence) reading or interpretation in this paper, which simply reflects the psycholinguistic viewpoint we shall adopt throughout. That is, the focus here will lie on the actual meanings hearers/readers compute when processing and comprehending compound sentences, the single interpretation that, everything else being equal, would match one of the true values of a connective's truth table. This choice of words is obviously not meant to replace the more common formulation employed in semantics when it comes to discussing the meaning of the connectives, according to which a given truth table specifies the situations or states of affairs that verify a given connective.

^2^We should note that we are not outlining a study that is, in some way, based on the distinction between conscious and unconscious mental processes, nor did we aim to study these kinds of processes as such; we are simply bringing attention to the different kinds of data—nay, the different sources of data—that we obtained in the experiments we ran, which were meant to prove (or disprove) our predictions, as explained below.

^3^Natural languages can also express the meanings of the unlexicalised connectives through paraphrases of various kinds, but this will not be our main concern here; we are focused on the lexicalisation of non-existent connectives, not on their verbalisation through means other than single words. In addition, it is important to advance here, as this be key later on, that NAND and NOR form a superset/subset relationship, as the truth table of NOR is a subset of the truth table of NAND, as can be seen on Table 1.

^4^It is worth noting that the principle of effability is about the expression of a sentence's underlying thought in different languages; the principle does not claim that it is possible to translate all the nuances of a message being communicated in one language into another language—as Keenan (1978) argues—be these syntactic, semantic, or else.

## Supplementary material

The Supplementary material includes a note clarifying the nature of the statistical analyses of the eye-movement data as well as the full details of the statistical summaries and the model comparisons that were conducted.
