# Supplementary material for: Unsaid thoughts: Thinking in the absence of verbal logical connectives
Source: Front Psychol. 2022 Sep 28;13:962099. doi: 10.3389/fpsyg.2022.962099 (PMC9554482; doi:10.3389/fpsyg.2022.962099)
Supplement: Supplementary file 1 [file Data_Sheet_1.pdf]

Supplementary Material

1 A NOTE ON ANALYSING EYE-MOVEMENT DATA

The analysis of eye-movement data can present some challenges in the study of cognition and there is at present no consensus on how best to approach this issue. As is usually the case, much depends on the actual design of an experiment. A study employing the *visual world paradigm* typically involves measuring participants' fixations to specific regions of a visual display given a specific stimulus; in a psycholinguistic study such as this one, the input stimulus is often a sentence describing some aspect of the scene that is presented to participants on a computer screen. In the case of a display showcasing 4 objects or graphics, the design used here, the screen can be divided into quadrants, or areas of interest (AOIs), where each graphic is placed on a quadrant and each AOI is classified according to how each graphic relates to the sentence played during the trial —viz., as the target interpretation for the sentence, as a competitor for the targeted interpretation, as a distractor, etc. (in most cases, a 4-way display exhibits one target, one competitor, and two distractors). In such a setting, the analysis is usually centred on whether the AOIs differ significantly on the likelihood of being fixated on, including when any such differences arise during the time course of a trial, with the dependent variable commonly analysed as "looks to the target AOI versus looks elsewhere" (where "looks" can be treated as proportions of fixations, log odds, binomial data, etc.). A number of techniques have been employed to inferentially test observed differences, but not without problems.

A widely-used way to analyse these data in the past was to compare proportions of fixations to different AOIs on specific time windows by using ANOVAs or t-tests (proportions of fixations are means of raw proportion scores, with a range between '0' and '1'). However, the use of ANOVAs and t-tests on time-series data can often violate certain assumptions of these statistical methods (Barr, 2008), and even when this is not the case, the comparisons may only unearth rather general patterns (e.g., whether a given comparison is significant in a time window of 500 milliseconds, for instance, may not be very informative). A better way to analyse eye-tracking data is to convert proportions of looks to binomial data —a fixation on a given AOI at a particular time would be coded as '1', or success, and '0' otherwise— and to employ a generalized linear mixed-effect model to run a logistic regression (Barr, 2008; Donnelly and Verkuilen, 2017). This can be complemented with orthogonal polynomials time functions to implement a so-called *growth curve analysis* (Mirman et al., 2008), which allows researchers to assess the predictive value of the experimental conditions over time (that is, the analysis can tell you which time polynomials interact with the conditions under investigation).

The design of our experiments presented additional analytical challenges and a *growth curve analysis* was not entirely appropriate for the purposes of the study; among other reasons, this kind of analysis does not reveal the precise moment in time where differences between AOIs become significant, and this is what we were actually interested in. As explained in the main text, in our experiments each AOI exhibited a unique combination of the truth values of the truth table of a logical connective (4 combinations to a truth table: True-True, True-False, False-True, and False-False) and thus each AOI was potentially relevant for the interpretation of the compound sentences participants would be exposed to. In addition, the length of the eye-tracking record of our experiments, a total of 6000 milliseconds per trial (though see below for a clarification), was likely to give rise to two problematic issues: that over such a long record fixations might exhibit a non-linear pattern, as indeed suggested by the wiggliness of the lines representing proportions of

fixations shown in Figure 1, Panel B, from the main text, a plot that was generated to visually inspect the data before analysis; and that there would be a significant amount of autocorrelation in the data, a common occurrence in experiments dealing with time-series (Baayen et al., 2018).

The autocorrelation issue was especially problematic in our study on account of some features of the experiments. In particular, the overall task was effectively underlain by a four-way, multinomial choice, as all AOIs were technically relevant and thus eye movements to all four AOIs needed to be tracked. In order to account for this aspect of the task, we recoded the probabilities of fixations to each AOI as binomial data, where '1' would mark a fixation on a specific AOI at a particular time, with the remaining three areas coded as '0' as non-fixations at that same time ("area of interest" was one of the predicting factors included in the analyses and constituted the indicator variable for the coding of the multinomial model). Crucially, this meant that the data had to be recoded on a millisecond by millisecond basis and thus at the sampling rate the eye tracker recorded the eye movements, whereas in most visual world studies the data are downsampled to 20 millisecond chunks prior to the analyses, which can decrease the amount of autocorrelation significantly, though not completely, and some information (or patterns) may be missed by so doing. In the case of our experiments, the amount of autocorrelation was bound to be very high and this was indeed confirmed in the analyses, as explained below.

We employed a *generalised additive mixed-model* (GAMM) (Wood, 2017) to account for these issues, a statistical technique that is becoming more common in analyses of time-series data (Baayen et al., 2017, 2018), including a few eye-tracking studies (Montero-Melis and Jaeger, 2019). GAMMs are especially useful on account of three features: the models relax the assumption of a linear relationship between predicting variables and response variables by implementing *smooth functions*, including so-called *factor smooths*, which can implement wiggly random effects (the non-linear equivalent of random intercepts and random slopes); autocorrelation can be accounted for by the inclusion of an autoregressive AR-1 parameter (in combination with factor smooths); and, particularly convenient for this study, the interpretation of the models is partly determined visually, allowing us to plot, among other things, the differences between conditions at specific moments in time.

The visual evaluation of GAMMs is very important and constitutes one of the three ways to test the significance of these models. The other two involve inspecting the summary statistics of each model and conducting model comparisons in terms of chi-square tests on both fREML scores and the difference in degrees of freedom specified in each model (model comparison in terms of AIC scores, the usual way to compare linear models is not reliable when an AR-1 parameter is included). We shall employ all three methods in the analyses below.

2 EXTENDED MATERIALS AND METHODS

Given that the present study was centred on the semantic and logical properties of compound sentences and the core objective was to probe the availability of the unlexicalised connective NAND, a number of issues required special attention. First of all, we used experimental materials that minimised some of the pragmatic effects that sometimes arise when interpreting such sentences, in line with a framework employed before to this effect (Lobina et al., forthcoming). This was potentially a factor in Experiment 1, where we used disjunctive sentences as the experimental condition and such sentences tend to elicit an exclusivity implicature, thereby countenancing the inclusive interpretation of disjunction. It is unclear whether similar effects would surface in Experiment 2, where we employed compound sentences in which the two clauses were connected by a made-up word standing for the NAND connective. In principle, participants have no

experience whatsoever with NAND sentences, and barring any problems regarding their ability to learn the meaning of a non-existent word for NAND during the learning phase of this experiment, no further, extraneous effects were expected or predicted.

The sentences we designed simply ascribed different colours to various geometrical figures, thus greatly restricting the context participants are offered as well as being rather neutral as to what interpretation would be favoured. An example of each condition from each experiment is presented below in Spanish, the language of the study (the translations appear in parentheses; note that in Experiment 2 we used the non-existent but possible Spanish word *fro* for the NAND connective; see below for more details).

Or condition, Experiment 1:El círculo es azul *o* el cuadrado es amarillo (the circle is blue OR the square is yellow)
NAND condition, Experiment 2:El círculo es azul *fro* el cuadrado es amarillo (the circle is blue NAND the square is yellow)

In addition, we took two different kinds of measurements in each trial —the participants' eye-movement record as the sentence was played, and behavioural responses at the end of each trial when participants were asked to select all the quadrants they thought matched the sentence they had heard— and it was hoped that in combination these data would provide a more comprehensive picture of the overall interpretation of the manipulated sentences. Participants might exhibit a preference for an exclusive interpretation of disjunction in their responses, for instance, but the eye-tracking data could potentially show what sort of (implicit) consideration they give to relevant other readings, and this was deemed to be of potential interest for NAND sentences as well (as proved to be the case, as shown below and discussed in the main text).

Finally, and as a way to make sure performance on the NAND condition would not be affected by the nature, or novelty, of the general set-up, participants were required to carry out both experiments in the same session (with various breaks), first Experiment 1 and then Experiment 2. It was thought that Experiment 1, which did not employ any non-existent words but language's disjunction *or*, would provide a gentle sort of introduction to the overall setting so that participants were sufficiently familiar to the task at hand by the time they were exposed to NAND sentences in Experiment 2. Further, disjunctive sentences were specifically chosen in order to avoid the contrast participants might have considered between the NAND connective and its contrary, conjunction *and* (see Table 1 from main text), which could have introduced an unfortunate artefact. Namely, by using conjunctive sentences participants might have realised that correctly interpreting NAND sentences simply required entertaining the opposite set of facts to conjunction, but no such comparison was possible between *or* and NAND (though there was an interesting point of contact between the two in relation to the unlexicalised connective NOR, the contrary of inclusive disjunction; we come back to this below).

2.1 Experiment 1

Participants. 15 psychology students (1 male, 14 female) from the Rovira i Virgili University (Tarragona, Spain) participated in this and the next experiment for course credit. None of the participants had undertaken any course in logic or reasoning before taking part in the experiments. All participants carried out Experiment 1 first, and after a short break, Experiment 2. The mean age was 18.5 years ($SD = 0.68$), and none of the participants had any known hearing or visual impairments. All were native speakers of Spanish. All participants gave their written informed consent before taking the experiment, the experiments followed the Rovira i Virgili University research and ethical guidelines, and the overall study itself was approved by the University's Ethics Committee on Research into People, Society, and the Environment (CEIPSA).

Materials. This experiment evaluated a single condition with 4 levels. Each condition corresponds to each of the four values of the inclusive disjunction's truth table (TTTF). 16 biclausal, declarative Spanish sentences, with the two clauses connected by the coordinator *or*, were constructed. Each clause ascribed a single colour to a single geometrical figure; we used four different colours (blue, yellow, red, and green) and four different figures (circles, squares, triangles, and diamonds). 16 more sentences were constructed to act as fillers. The fillers were monoclausal and thus only one figure was mentioned, but in this case the figure was ascribed two colours instead of one by employing the connective *and* and the overall sentence was furthermore negated (e.g., *the circle is not blue and green*). A further 8 practice sentences were created, four of which were similar to the experimental items and four to the fillers. The average length of all sentences was 2555 milliseconds (ms) and the longest sentence was around 3000 ms. The sentences were recorded in stereo with a normal but subdued intonation by a native, male speaker of the Spanish language using the Praat software on a Windows-operated computer. The graphics representing each one of the truth values of inclusive disjunction as well as the truth values of negated conjunctions (for the fillers) were created with Microsoft PowerPoint.

Procedure. The experiment was designed and run with the Experimental Builder software (SR Research Ltd.) and administered in a laboratory with low to normal illumination in which each participant was tested individually. Participants were seated in front of a computer screen and were asked to place their head on a chin rest. The chin support was adjusted for each participant so that there was a distance of around 60 centimetres between their eyes and the monitor where the visual scene was presented, a 19-inch screen set to a resolution of 1024 × 768 pixels. The position and fixations of participants' right eye, most people's dominant eye, were continuously recorded at a sampling rate of 1000 Hz with an EyeLink 1000 eye tracker. In addition to the eye-tracking data, the participants' behavioural responses at the end of the trials were also recorded.

The overall flow of the experiment as well as the general design is shown in Figure 1, Panel A, from the main text. Each trial started with a fixation point in the middle of a white screen. Participants were asked to fixate on this point and to press the space bar when they were ready to start the trial. A sentence such as *the figures will be triangles and squares* would replace the fixation point 500 ms after pressing the space bar. The sentence would stay on the screen for 2500 ms so that participants had enough time to read it fully; at the end of this period of time, the sentence was replaced by the visual display, which would remain on the screen for the remainder of the trial. The display was divided into quadrants and a specific combination of figures and colours would appear on each quadrant, where the figures matched those announced in the sentence presented before the visual display (the placement of each graphic was randomised across quadrants and trials). After 2000 ms, the time we allocated to participants to view the quadrants fully before presenting any other stimuli, a sentence describing one or more quadrants was played over headphones binaurally. Once the sentence had finished, there was a period of 3000 ms of "looking time", at the end of which the cursor would be activated so that participants could select the quadrants they thought the sentence described appropriately. The trial ended once participants were satisfied with their answers and had pressed the space bar to move on to the next trial (or reach the end of the session). Participants carried out an 8-item practice session with the experimenter, who explained the overall task and answered any questions before proceeding to the experimental session. Eye calibration was conducted before the practice session and again before the experimental session. The experimental session consisted of a total of 32 items, 16 of which were experimental and 16 fillers; the presentation of experimental and filler sentences was randomised. The experiment lasted 15 minutes overall.

Results. *Eye-tracking data*. The eye gaze data collected with the EyeLink 1000 eye tracker was exported by using the manufacturer's Data Viewer software. The Sample Report this software outputs requires significant preprocessing before analysis and plotting, and we used the R package *VWPre*, version 1.2.4, for this purpose (Porretta et al., 2016). To begin with, we performed an analysis of trackloss (i.e., the amount of times the eye tracker lost track of participants' eye gaze); 1.68% of data was marked as off-screen and 4.86% as trackloss, and as a result 7 trials with less than 75% of data were eliminated (this threshold is common in the literature and seemed reasonable for our own experiments too). The data were then prepared in order to conduct a logistic GAMM analysis. As the task was effectively underlain by a four-way, multinomial choice and thus all AOIs were potentially valid interpretations for the sentences, the factor "area of interest" (AOI) was the main predictor in our models and constituted the indicator variable for the coding. The data were coded on a millisecond by millisecond basis, where a fixation on a given AOI at a given time was coded as a '1' (for success) and a non-fixation as a '0' (for failure).

The logistic GAMMs were conducted and partly analysed with the R package *mgcv*, version 1.8-35, family class binomial (Wood, 2017). Three such models were run and compared, and their analysis was complemented with the R package *itsadug*, version 2.4 (van Rij et al., 2020). We used model selection to determine the best random-effects structure for the data, which is the most appropriate approach for non-linear models such as GAMMs (Baayen et al., 2017; Wieling, 2018). The R script for the analyses of this and the next experiment as well as the eye-tracking data can be found at: `https://osf.io/mfqt8/`.

An important factor to consider when using the *mgcv* package is that fitting GAMMs often requires significant computational resources and some processes may take a very long time to complete, in some cases even days (fitting such models certainly takes much longer than fitting linear mixed-effects models with the *lme4* package, a staple of contemporary research). Given that in the 6000 ms of eye movements we recorded in each trial a pattern was pretty evident after 4000 ms and did not change in any way after that, as shown in Figure 1, Panel B, from the main text, for both experiments, we decided to reduce the length of the time-series to analyse to 4000 ms, which was hoped would ease the demands of the analyses. The average length of the experimental sentences from Experiment 1 was 2947 ms and this meant that the models we analysed included at least 1000 ms of "looking time", which was abundantly sufficient for the purposes at hand and thus no important effects were expected to be missed.

Three models were run, one of which, M0, was unlikely to fit the data very well and was primarily used to estimate the amount of autocorrelation to correct in the models we expected to fit the data better, models M1 and M2. In all models, the AOI variable was fitted to the response variable IsFixated, which as discussed earlier was treated as a binary kind of data ('1' for a fixation on an AOI at a given time, '0' otherwise). All three models included a smooth function for Time by AOI (TT, TF, FT, and FF; the function appears as *s(Time)* in the model itself, as shown in Table S2), allowing us to assess whether there was a non-linear relationship between the fixed-effect AOI condition and the response variable IsFixated over time (that is, this smooth function assessed the one-dimensional —there is only one numerical predictor— interaction between the factors Time and AOI). Regarding the random effects, model M0 was fitted with by-participants and by-items random intercepts and slopes (4 curves in total), which were not expected to capture the variability very well given the potential non-linearity of the data. A visual inspection of the autocorrelation, shown in Figure S1, Top Panel, confirmed the high amount of autocorrelated data and furthermore indicated that there was only a slight decrease in such autocorrelation across the time course of trials (the decrease is more marked in downsampled, 20 ms chunks (van Rij et al., 2019)). Following the general practice from the literature, we took the amount of autocorrelation at time lag 1, $\rho = 0.995$, to be an appropriate measure for the amount of autocorrelation to be corrected (Baayen et al., 2018). M0 was rerun

with an AR-1 parameter and the autocorrelation was eliminated, as shown in Figure S1, Bottom Panel (this was the case for every other model we ran and thus abstain from adding any more graphics on this issue). The summary statistics of model M0 indicated that its random structure did not account for much of the variability (only one of the four curves was statistically significant) and thus we disregarded this random structure from subsequent models (further, a comparison of the summary statistics of a model that included an AR-1 parameter with a model that did not include this parameter illustrates the unreliability of models that do not control for autocorrelated data; these summary statistics are not included here, however).

Models M1 and M2 were set up to account for non-linear random effects, though in different ways, as we shall see. What these models did share, as mentioned, was a smooth function for Time by AOI as well as an AR-1 parameter to correct the autocorrelation, and to this were added functions to model possible trial effects, one of the "human factors" the field is ever more concerned with (Baayen et al., 2017). In particular, we added a smooth function for Trial by AOI (*s(Trial)* in the model) to assess the (one-dimensional) interaction between trial number and AOI, and a tensor product to evaluate the two-dimensional interaction between the factors time and trial number by AOI (this interaction is two-dimensional because there are two numerical predictors to compare, one for the Time factor and one for Trial Number factor; the tensor product itself appears as *ti(Time, Trial)* in the model). As for the random effects, M1 was fitted with by-participant and by-item factor smooths for Time by AOI, replacing the random intercepts and slopes from M0 and added to this model in order to implement wiggly random effects (factor smooths are centred and in fact penalised against non-linearity), while M2 included factor smooths for Time per time-series by AOI (i.e., per trial; *s(Time, Event)* in the model), replacing the factor smooths from M1 and potentially offering a closer fit to the data ("event" smooths tell the model that the measurements for each time series are not in fact independent, a feature that ought to decrease the amount of autocorrelation as well). All together, model M1 yielded 20 parameters (or curves) and model M2 a total of 16. In order to evaluate these models, we start with the summary statistics, followed by model comparison and then, crucially, as we shall point out, a visual evaluation of the best-fit model.

For every smooth function of M1 and M2, except for the smooth for Trial, non-linear curves were obtained, as confirmed by the effective degrees of freedom (edf), a summary statistic of GAMMs that reflects the degree of non-linearity of a curve. An edf equal or close to 1 corresponds to a linear relationship between the predicting variables and the response variable, and anything above 2 equals to a highly non-linear relationship, which is what was observed in the two models for every smooth except, as alluded to, for the effect of Trial by AOI (with a $p < .001$ in every case a non-linear relationship was observed). As for the Time x Trial interaction, there was a significant effect regarding the TT AOI ($p = .044$ in M1, $p = .028$ in M2). In such circumstances, the predictor Trial by AOI would normally be eliminated from the models, but a GAMM that includes a tensor product to evaluate a two-dimensional interaction assumes that there is a smooth function for the first factor of the interaction (here, Time) and a smooth function for the second factor (here, Trial Number), and therefore the smooth for Trial by AOI was kept in the final analyses (GAMMs also allow modelling a type of tensor product that implicitly includes the smooths for each of the two factors being compared, a so-called *te*-operator, but this would not have been conducive to our objectives, as we needed to directly asses the significance of the smooth function for Time by AOI, the fixed effect).

Regarding model comparison, we used a Maximum Likelihood (ML) score comparison, which is preferred over a comparison in terms of AIC scores, as AICs become unreliable (i.e., anti-conservative) when autocorrelation is included in a model. Table S1 shows the details of the comparison between M1 and M2, where the differences in fREML scores and degrees of freedom indicate a preference for model

M2, the model with event factor smooths instead of by-participants and by-items factor smooths. Table S2, in turn, provides the full summary statistics of the best-fit model, M2 (note the values of the various edfs as well as the p-values for each one of the non-linear curves).

As for the visual inspection of the best-fit model, three graphics are relevant in this case. Figure S2 shows the fixations on each quadrant according to M2, and a useful comparison can be drawn between the response variable IsFixated from this graphic to the proportions of fixations from Figure 1, Panel B, from the main text. Two-dimensional interactions also require visual evaluation in GAMMs; Figure S3 provides a contour surface of the predicted fixations on the TT quadrant for the interaction between Time and Trial Number. As can be seen in the graphic, the likelihood that the TT quadrant is fixated on generally increases in time during each trial, but at 4000 ms fixations on the TT quadrant are less likely as the experimental session progresses —that is, the likelihood that the TT quadrant is fixated on actually decreases from trial 20 onwards towards the end of the time series, as indicated by trial number on the y-axis (these numbers refer to all items, but the data from the graphic only includes the fixations on the experimental items). This effect is rather significant given that model M2 included event smooths, which could have captured many of the effects to do with trial number.

In turn, and rather importantly, Figure 2, Panel A, from the main text, shows the "difference curves" from M2 between the smooth function for TT, the most fixated AOI, against the remaining three smooths (TF, FT, FF), where the relevant time windows are marked —that is, the graphics show the estimated differences between fixations to the TT AOI and fixations to the TF, FT, and FF AOIs. These curves as well as the accompanied statistics are implemented by the package *itsadug* and constitute the most relevant way to assess whether the different levels of the AOI condition significantly differ from each other in this experiment (GAMMs allow other ways to probe whether the different levels of an experimental condition differ from each other, such as by employing ordered or binary factors, but we put this issue to one side here). In particular, the graphs of Figure 2, from the main text, show the comparison between the (non-linear) smooth of the AOI with the most fixations (fixations are labelled as IsFixated on the y-axis) against each of the (non-linear) smooths of the other AOIs, with the gray solid line indicating the estimated difference. The shaded band represents the pointwise 95%-confidence interval; when the band doesn't overlap with the x-axis (i.e., the value is significantly different from zero), this is indicated by a red solid line on the x-axis along with red vertical dotted lines.

The graphs show fixations during the audio of the entire sentence in addition to an extra 1000 ms of looking time. The TT-TF comparison exhibits differences in two time windows, at 445–930 ms, around the time the first clause is being played in the audio, and at 2750–3999 ms, where the beginning of this time-window coincides with the end of the audio. The TT-FT contrast produces a difference at 1250–3999 ms, a window that (roughly) starts right after the connective has been presented, while the TT-FF comparison exhibits a difference at 890–3999 ms, where the beginning of this time-window precedes the presentation of the connective.

Behavioural responses. Regarding the behavioural data, presented in Figure 1, Panel C, from the main text, the preferred pattern of response for disjunction *or*, shown on the table on the left-hand side, was TTTF —that is, participants selected the TT, TF, and FT quadrants— in line with the truth table of logic's inclusive disjunction. For the analysis of these data we drew a distinction between acceptable (or correct) patterns of response and unacceptable (or incorrect) patterns in order to run chi-squared tests between the expected and observed responses, in two steps: first between acceptable and unacceptable responses as a way to confirm that the sentences had been interpreted correctly, and then within acceptable responses between the two patterns of interpretation we had identified beforehand as being correct. In this experiment,

we took the patterns TTTF and FTTF to be the only acceptable responses, as these constitute the truth tables of inclusive and exclusive disjunction, respectively, and thus everything else was regarded as a mistake in interpretation. The percentage of correct answers amounted to 67.41, for 32.59 of incorrect answers, and the difference between these two frequencies was significant ($\chi^2(1) = 27.16, p < .001$). Once it was ascertained that the task had been appropriately carried out by the participants, we compared the two patterns of acceptable responses. In this case, the percentage of responses for the inclusive interpretation of disjunction (TTTF) was 80.79, for 19.21 for the exclusive interpretation (FTTF), and this difference was clearly significant as well ($\chi^2(1) = 57.29, p < .001$).

2.2 Experiment 2

Participants. The same as in Experiment 1, as noted above.

Materials. This experiment also evaluated a single experimental condition with 4 levels, but in this case the two clauses of the compound sentences were connected by a non-existent but possible word in Spanish standing for the unlexicalised logical connective NAND (truth table: FTTT): *fro*. The nonsense word *fro* is not related to, nor does it resemble, any other word in Spanish (or Catalan, the experiment took place in Catalonia), it is easy to pronounce, and its morphology favours the sort of use participants would be exposed to in the experiment (i.e., as a coordinator). We used the same figures and colours employed in Experiment 1, and a total of 32 NAND sentences were constructed, 16 experimental sentences and 16 sentences meant for the learning phase. As for the filler sentences, these were also similar to those of Experiment 1, but in this case the monoclausal sentences were not negated. A further 8 practice sentences were created, four of which were similar to the experimental items and four to the fillers. The average length of all sentences was 2837 ms and the longest sentence was around 3500 ms. All other details remained the same as in Experiment 1, except that that the graphics for the experimental sentences now represented each one of the values of the truth table for NAND and the graphics for the fillers the values of the truth table for (non-negated) conjunctive sentences.

Procedure. The procedure was very similar to that of Experiment 1, with the addition of a learning phase for the connective NAND. In this phase, which was undertaken before the practice session, participants would be shown a series of situations that NAND sentences could appropriately describe (or not). In particular, participants would be exposed to individual graphics in each trial, with each graphic always depicting two geometrical figures in two different colours, as in the experimental materials. The rationale was that participants would be exposed to individual graphics in each trial so that each one of the truth values from the truth table for NAND would be presented individually. Regarding the actual procedure of the learning phase, the graphic would appear on the screen first, and after a brief period of time for participants to inspect it adequately, a NAND sentence would be played over the headphones. Soon after participants would be presented with a feedback on screen indicating whether the sentence was an appropriate description of the graphic or not —with a tick, for 'yes', and an X for 'no'. Participants undertook four iterations of the truth table for NAND, for a total of 16 trials; that is, the four values of the truth table of NAND —viz., FTTT— were repeated four times so that participants were exposed to 16 different NAND sentences (the order of presentation was randomised). There was no explicit instruction of any kind, nor did participants have to complete any task; instead, the learning phase was similar to what is employed in artificial grammar learning studies (Fitch and Friederici, 2012) and the expectation was that participants would implicitly learn the meaning of the novel word being presented to them given a specific set of scenes (a similar strategy is usually employed in studies of language acquisition (Akhtar and Tomasello, 1997)). Once this phase had been completed, participants undertook an 8-item practice session

and right after an experimental session of 32 items (16 experimental sentences, 16 filler sentences); all other aspects of the experiment remained the same as in Experiment 1 (4 areas of interest per trial, randomisation, etc.). Eye calibration was conducted before the practice session and again before the experimental session, but it was not included in the learning phase. This experiment lasted around 20 minutes overall.

Results. *Eye-tracking data:* The eye gaze data was prepared in the same way as the data from Experiment 1 was prepared. In this case, an analysis of trackloss marked 1.83% of data as off-screen and 6.08% as actual trackloss, and thus 11 trials with less than 75% of data were eliminated. In this experiment too the time series were reduced to 4000 ms and this yielded around 640 ms of looking time (the average length of experimental sentences was 3366 ms), which was also amply sufficient for the analyses, as the overall pattern of this experiment was in fact established significantly early, around 1400 ms. GAMMs were processed and analysed in the same way as in Experiment 1, and the same three kinds of models were run —that is, the details of models M0, M1, and M2 were exactly like the corresponding models from Experiment 1. In this experiment, the amount of autocorrelated data was estimated at $\rho = 0.996$ after running the equivalent M0 model, and in this case too this model was discarded because its random structure failed to capture the data adequately.

Models M1 and M2 from this experiment were evaluated in the same way that the models from Experiment 1 had been evaluated. In terms of summary statistics, there were hardly any trial effects in either model, in terms of either the smooth function for Trial by AOI (expect for one borderline case; see Table S4) or the tensor product for the Time x Trial interaction. In all other cases, the smooth functions resulted in non-linear curves in every case, with all edfs above 2 and $ps < .001$. Model comparison favoured model M1 over model M2, as shown in Table S3, where the chi-square statistic indicates a significant difference in likelihood between the models, given fREML scores and the degrees of freedom, and the p-value suggests that there is strong evidence in favour of model M1. That is, model M1, which included by-participants and by-items factor smooths —*s(Time, Subject)* and *s(Time, Item)* in the model from Table S4, respectively— was preferred to model M2, which included event smooths. Thus, and though a smooth for Time per time-series (per event) is usually expected to account for the random structure better than the by-participants and by-items factor smooths, as had been the case for the best-fit GAMM for disjunction *or*, this did not prove to be so in this case.

Regarding the visual inspection of model M1, two graphics are relevant. Figure S4 shows the fixations on each quadrant according to M1, and once again it may be useful to compare the IsFixated, binomial data from this graphic to the proportions of fixations from Figure 1, Panel B, from the main text. Figure 2, Panel B, from the main text, in turn, shows the model's difference curves, or estimated differences, between fixations to the FF AOI and fixations to the FT, TF, and TT AOIs. Comparisons show that all differences surfaced after around 1400 ms, when the connective NAND had already appeared and the second clause of the sentences was being played.

Behavioural responses: The analysis of the behavioural data of this experiment presented a slightly more nuanced state of affairs than had been the case in Experiment 1. Whilst the full interpretation of the NAND connective would correspond to the FTTT pattern —that is, the quadrants TF, FT, and FF, which was the most frequent response, in fact— the FFFT response (that is, only quadrant FF is true), properly speaking the truth table of the (unlexicalised) connective NOR, the contrary of inclusive disjunction, cannot be regarded as an entirely incorrect or unacceptable response for NAND, but perhaps simply an incomplete one. As noted, NAND is the opposite of conjunction, and in this sense, the FF interpretation is plausibly the most straightforward counterpart to the TT reading of conjunction —or the default, preferred interpretation, as the eye-movement data actually seem to indicate. Indeed, participants overwhelmingly fixate on the

FF quadrant, which on its own may have indicated that participants understood the NAND connective as actually the NOR connective (as is the case in the relationship between inclusive and exclusive disjunctions, the true values of NOR's truth table constitute a subset of the true values of NAND's truth table). As such, we included FFFT as the second acceptable response along with FTTT. Thus established, the overall percentage of acceptable responses was a total of 64.73, for 35.27 of unacceptable answers, and the difference between the two frequencies proved to be significant ($\chi^2(1) = 19.45, p < .001$), confirming that NAND sentences were interpreted correctly. And within acceptable answers only, 82.07 percent of responses corresponded to the FTTT pattern and 17.93 percent to the FFFT response, and the difference was clearly significant here too ($\chi^2(1) = 59.65, p < .001$).

3 FIGURES AND TABLES

3.1 Figures

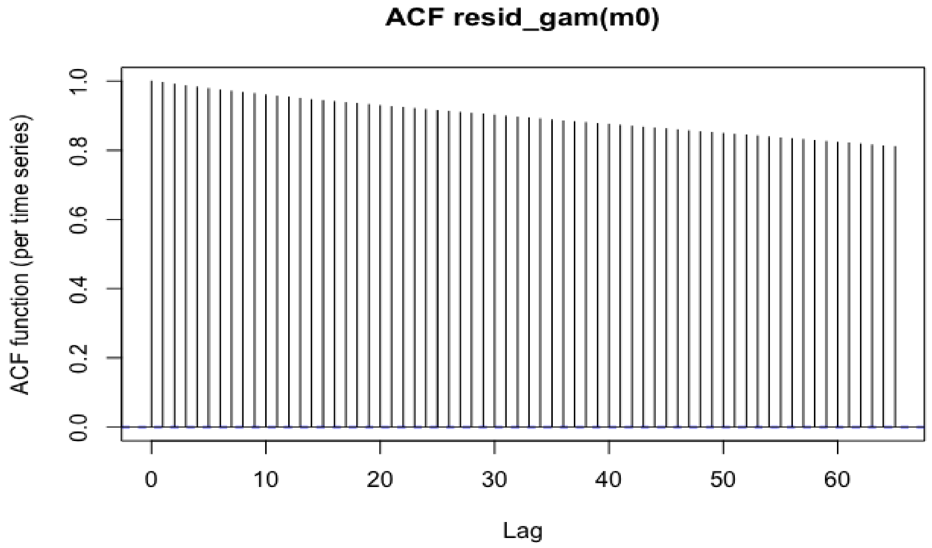

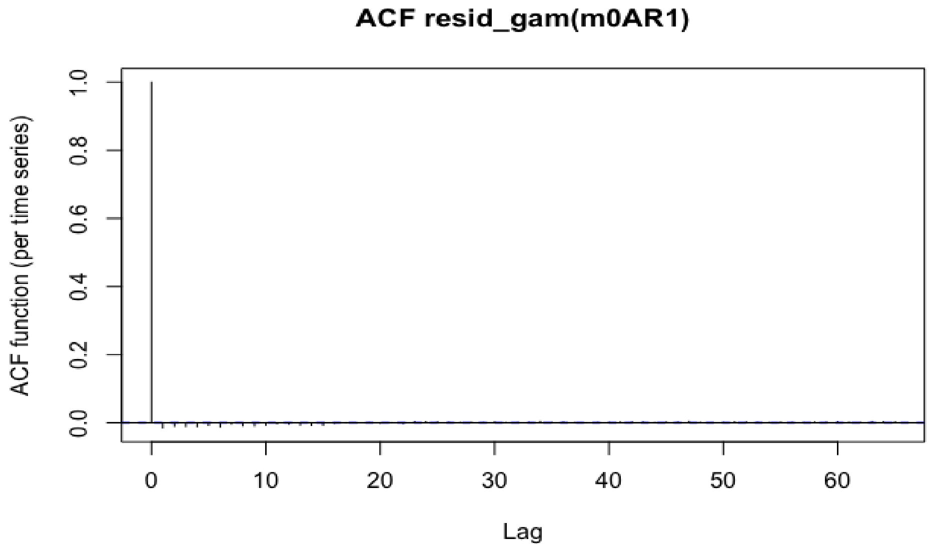

Figure S1: Amount of autocorrelation in two versions of model M0. Top panel shows the autocorrelation in a model without an AR-1 parameter (m0), while bottom panel shows the autocorrelation in a model with an AR-1 parameter (m0AR1). The autocorrelation is naturally ‘1’ at time lag 0 (i.e., each point has a correlation of 1 with itself), decreasing therefrom. The height of the second line indicates the amount of autocorrelation at lag 1.

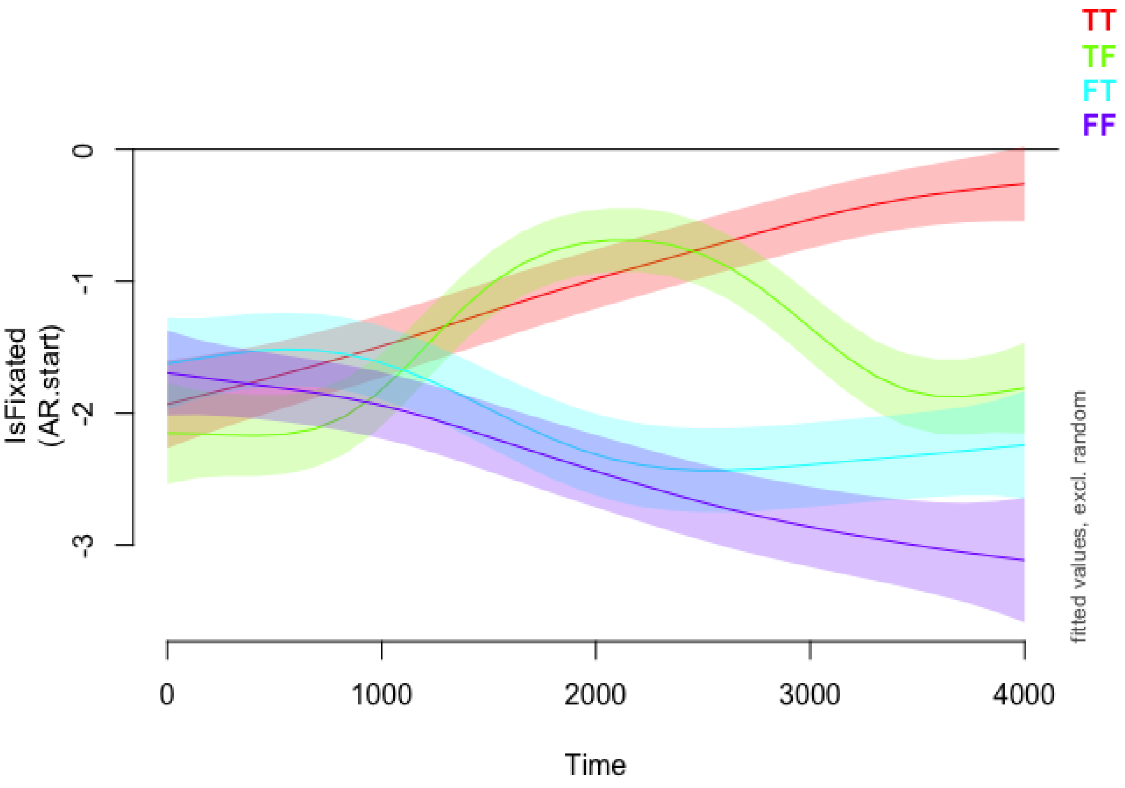

Figure S2: Non-linear smooths (fitted values) for fixations to each quadrant (TT, TF, FT, and FF) of model M2, Experiment 1, for a duration of 4000 milliseconds. The pointwise 95%-confidence intervals are shown by shaded bands.

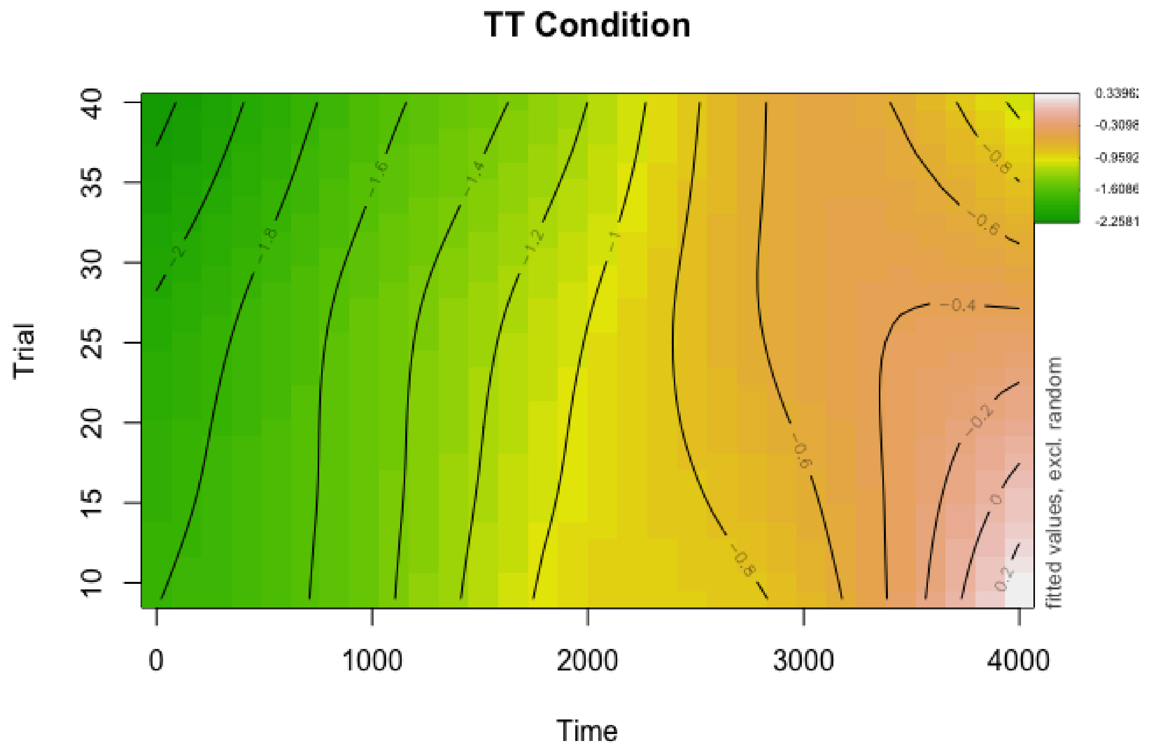

Figure S3: Contour surface for the Time x Trial interaction on the TT condition from model M2, Experiment 1. As the legend on the top-right corner states, areas in green indicate a smaller likelihood that the TT condition is fixated on, while a change of hue from green to yellow and then to orange indicates an increase in likelihood for fixations on the TT condition. Thus, there is an increase in the likelihood that the TT quadrant is fixated on across time, though towards the end of the time-series there is an actual decrease in the likelihood that the TT quadrant is fixated on as the experimental session progress —that is, there is a decrease in likelihood from trial number 20 onwards towards the end of each trial.

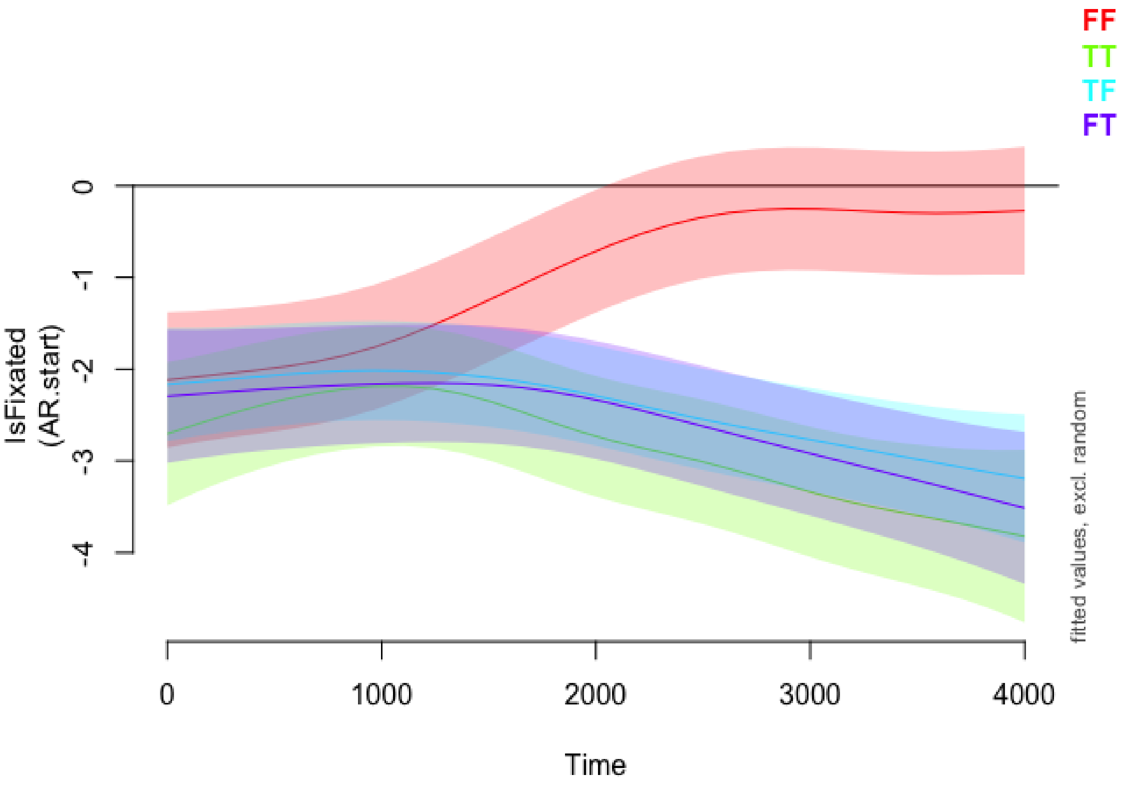

Figure S4: Non-linear smooths (fitted values) for fixations to each quadrant (TT, TF, FT, and FF) of model M1, Experiment 2, for a duration of 4000 milliseconds. The pointwise 95%-confidence intervals are shown by shaded bands.

3.2 Tables

Table S1. Maximum Likelihood comparison between models M1 and M2, Experiment 1. M2 is preferred: lower fREML score (222582.690) and lower df (8.000).

Model	Score	Edf	Difference	Df
M1	-3587391	48	NA	NA
M2	-3809974	40	-222582.690	8.000

Table S2. Model Summary for M2, Experiment 1, reporting parametric coefficients (Part A) and estimated degrees of freedom (edf), reference degrees of freedom (Ref.df), F-values, and p-values for smooth terms (Part B).

A. parametric coefficients	Estimate	Std. Error	t-value	p-value
(Intercept)	-1.0590	0.0937	-11.3061	$<$ .001
AOITF	-0.4270	0.1289	-3.3117	$<$ .001
AOIFT	-1.0331	0.1329	-7.7732	$<$ .001
AOIFF	-1.3615	0.1406	-9.6826	$<$ .001
B. smooth terms	edf	Ref.df	F-value	p-value
s(Time):AOITT	2.5717	3.2734	77.3748	$<$ .001
s(Time):AOITF	7.0725	8.2467	131.2745	$<$ .001
s(Time):AOIFT	5.5059	6.8240	32.3071	$<$ .001
s(Time):AOIFF	3.5746	4.6142	45.7139	$<$ .001
s(Trial):AOITT	1.0163	1.0215	1.1134	.434
s(Trial):AOITF	1.0979	1.1349	0.0486	.822
s(Trial):AOIFT	1.2755	1.3917	2.3753	.129
s(Trial):AOIFF	1.0312	1.0468	1.1054	.317
ti(Time,Trial):AOITT	3.1402	3.6852	8.8481	.028
ti(Time,Trial):AOITF	1.0841	1.1605	0.0124	.981
ti(Time,Trial):AOIFT	1.4577	1.7883	3.7415	.217
ti(Time,Trial):AOIFF	1.0819	1.1612	0.5425	.525
s(Time,Event):AOITT	313.8023	1952.0000	1429.0621	$<$ .001
s(Time,Event):AOITF	182.2498	1951.0000	2070.8300	$<$ .0001
s(Time,Event):AOIFT	199.0825	1951.0000	951.4488	$<$ .001
s(Time,Event):AOIFF	101.6644	1951.0000	98240.7144	$<$ .001

Table S3. Maximum Likelihood comparison between models M1 and M2, Experiment 2. Chi-square test of fREML scores.

Model	Score	Edf	Difference	Df	p-value	Sig.
M2	-3800026	40	NA	NA	NA	NA
M1	4007992	48	207966.698	8.000	$< 2e - 16$	***

Table S4. Model Summary for M1, Experiment 2, reporting parametric coefficients (Part A) and estimated degrees of freedom (edf), reference degrees of freedom (Ref.df), F-values, and p-values for smooth terms (Part B).

A. parametric coefficients	Estimate	Std. Error	t-value	p-value
(Intercept)	-1.0634	0.3137	-3.3896	$< .001$
AOITT	-1.7535	0.4345	-4.0355	$< .001$
AOITF	-1.3671	0.4000	-3.4181	$< .001$
AOIFT	-1.3437	0.4137	-3.2476	.0012
B. smooth terms	edf	Ref.df	F-value	p-value
s(Time):AOIFF	4.8975	5.9350	59.5571	$< .001$
s(Time):AOITT	4.2796	5.3850	32.6855	$< .001$
s(Time):AOITF	2.9593	3.6781	12.9396	.009
s(Time):AOIFT	3.0391	3.7723	14.4254	.005
s(Trial):AOIFF	2.6887	3.3426	6.0813	.136
s(Trial):AOITT	1.2864	1.5064	1.7189	.212
s(Trial):AOITF	1.0488	1.0951	0.4639	.553
s(Trial):AOIFT	4.0588	4.9972	11.4329	.045
ti(Time,Trial):AOIFF	1.0466	1.0918	0.1404	.781
ti(Time,Trial):AOITT	2.4493	3.2051	3.2910	.402
ti(Time,Trial):AOITF	1.0954	1.1873	0.4488	.616
ti(Time,Trial):AOIFT	1.5503	1.9095	0.7982	.700
s(Time,Subject):AOIFF	43.2222	125.0000	181.1185	$< .001$
s(Time,Subject):AOITT	11.3449	125.0000	42.7670	$< .001$
s(Time,Subject):AOITF	11.0574	126.0000	48.2295	$< .001$
s(Time,Subject):AOIFT	19.5463	125.0000	68.6718	$< .001$
s(Time,Item):AOIFF	28.1990	143.0000	62.8804	$< .001$
s(Time,Item):AOITT	31.5330	143.0000	99.6503	$< .001$
s(Time,Item):AOITF	43.6394	144.0000	111.7409	$< .001$
s(Time,Item):AOIFT	40.7337	144.0000	100.4008	$< .001$

REFERENCES

Akhtar, N. and Tomasello, M. (1997). Young children's productivity with word order and verb morphology. *Developmental psychology* 33, 952–65

Baayen, R. H., van Rij, J., de Cat, C., and Wood, S. (2018). Autocorrelated errors in experimental data in the language sciences: Some solutions offered by generalized additive mixed models. In *Mixed-Effects Regression Models in Linguistics*, eds. D. Speelman, K. Heylen, and D. Geeraerts (Cham, Switzerland: Springer International Publishing AG). 49–70

Baayen, R. H., Vasishth, S., Kliegl, R., and Bates, D. (2017). The cave of shadows: Addressing the human factor with generalized additive mixed models. *Journal of Memory and Language* 94, 206–234

Barr, D. J. (2008). Analyzing 'visual world' eyetracking data using multilevel logistic regression. *Journal of Memory and Language* 59, 457–474

Donnelly, S. and Verkuilen, J. (2017). Empirical logit analysis is not logistic regression. *Journal of Memory and Language* 94, 28–42

Fitch, W. T. and Friederici, A. (2012). Artificial grammar learning meets formal language theory: an overview. *Philosophical Transactions of the Royal Society B — Biological Sciences* 367, 1933–1955

Lobina, D. J., Demestre, J., García-Albea, J. E., and Guasch, M. (forthcoming). Default meanings: Language's logical connectives between comprehension and reasoning. *Linguistics and Philosophy*

Mirman, D., Dixon, J. A., and Magnuson, J. S. (2008). Statistical and computational models of the visual world paradigm: Growth curves and individual differences. *Journal of Memory and Language* 59, 475–494

Montero-Melis, G. and Jaeger, T. F. (2019). Changing expectations mediate adaptation in l2 production. *Bilingualism: Language and Cognition* 23, 602–617

[Dataset] Porretta, V., Kyröläinen, A.-J., van Rij, J., and Järvikivi, J. (2016). VWPre: Tools for preprocessing visual world data. Version 1.2.4, updated 2020-11-28

van Rij, J., Hendriks, P., van Rijn, H., Baayen, R. H., and Wood, S. N. (2019). Analyzing the time course of pupillometric data. *Pupillometry in Hearing Science* 23, 1–22

[Dataset] van Rij, J., Wieling, M., Baayen, R. H., and van Rijn, H. (2020). itsadug: Interpreting time series and autocorrelated data using gamms. R package version 2.4

Wieling, M. (2018). Analyzing dynamic phonetic data using generalized additive mixed modeling: A tutorial focusing on articulatory differences between l1 and l2 speakers of english. *Journal of Phonetics* 70, 68–116

Wood, S. N. (2017). *Generalized Additive Models: An Introduction with R* (London, England: Chapman and Hall/CRC), 2 edn.
